# Thbs1 induces lethal cardiac atrophy through PERK-ATF4 regulated autophagy

**DOI:** 10.1038/s41467-021-24215-4

**Published:** 2021-06-24

**Authors:** Davy Vanhoutte, Tobias G. Schips, Alexander Vo, Kelly M. Grimes, Tanya A. Baldwin, Matthew J. Brody, Federica Accornero, Michelle A. Sargent, Jeffery D. Molkentin

**Affiliations:** 1 Department of Pediatrics, University of Cincinnati, Cincinnati Children’s Hospital Medical Center, Cincinnati, OH USA; 2 Department of Pharmacology, University of Michigan, Ann Arbor, MI USA; 3 Department Physiology and Cell Biology, The Ohio State University, Columbus, OH USA; 4 Howard Hughes Medical Institute, Cincinnati Children’s Hospital Medical Center, Cincinnati, OH USA; 5 Present Address: Janssen Pharmaceuticals, Spring House, PA USA

**Keywords:** Autophagy, Chaperone-mediated autophagy, Cardiology

## Abstract

The thrombospondin (Thbs) family of secreted matricellular proteins are stress- and injury-induced mediators of cellular attachment dynamics and extracellular matrix protein production. Here we show that Thbs1, but not Thbs2, Thbs3 or Thbs4, induces lethal cardiac atrophy when overexpressed. Mechanistically, Thbs1 binds and activates the endoplasmic reticulum stress effector PERK, inducing its downstream transcription factor ATF4 and causing lethal autophagy-mediated cardiac atrophy. Antithetically, *Thbs1*^*−/−*^ mice develop greater cardiac hypertrophy with pressure overload stimulation and show reduced fasting-induced atrophy. Deletion of Thbs1 effectors/receptors, including ATF6α, CD36 or CD47 does not diminish Thbs1-dependent cardiac atrophy. However, deletion of the gene encoding PERK in Thbs1 transgenic mice blunts the induction of ATF4 and autophagy, and largely corrects the lethal cardiac atrophy. Finally, overexpression of PERK or ATF4 using AAV9 gene-transfer similarly promotes cardiac atrophy and lethality. Hence, we identified Thbs1-mediated PERK-eIF2α-ATF4-induced autophagy as a critical regulator of cardiomyocyte size in the stressed heart.

## Abstract (web summary)

Beneficial and detrimental effects have been ascribed to the different Thrombospondin (Thbs) proteins in the adult mammalian heart. Here, the authors show that Thbs1-mediated activation of PERK-eIF2α-ATF4-induced autophagy regulates adult cardiomyocyte size in the stressed heart.

## Introduction

Cardiomyocytes within the adult mammalian heart are refractory to proliferation, hence all changes in heart size in response to increased workload, unloading, stress, or disease stimulation occur through hypertrophy or atrophy of individual cardiomyocytes^1^. Effector pathways central to size change of cardiomyocytes include calcineurin/nuclear factor of activated T-cells, mitogen-activated protein kinases, the mechanistic target of rapamycin (mTOR)/protein kinase B (AKT)/Forkhead box O (FOXO) pathway, Atrogin-1/MuRF1, and autophagy. These pathways regulate protein turnover by balancing protein synthesis and protein degradation and in general, hypertrophic growth takes place when protein synthesis outweighs degradation, and atrophy occurs when degradation outpaces new protein synthesis^2,3^.

The endoplasmic reticulum (ER) is the site where secreted or membrane bound proteins are generated, and this subcellular compartment dynamically responds to stress signals in regulating total protein production^4^. Accumulation of misfolded proteins in the ER also activates a signaling cascade known as the unfolded protein response (UPR), or simply the ER stress response. Unfolded ER proteins or stress are indirectly detected by protein kinase R (PKR)-like ER kinase (PERK), activating transcription factor 6α (ATF6α), and inositol requiring enzyme 1 alpha (IRE1α)^4^. Activation of these three primary sensors results in downregulation of general protein synthesis while also inducing expression of ER resident chaperones to enhance protein folding capacity^4^. Autophagy is induced by select signals that degrade misfolded proteins via lysosomes to also help resolve proteotoxic stress and to provide metabolic reserve^5^. The PERK-eukaryotic translation initiation factor 2 α (eIF2α)-activating transcription factor 4 (ATF4) pathway is essential in detecting ER stress and the induction of autophagy to resolve accumulation of misfolded proteins via lysosomal degradation^6,7^.

The family of thrombospondin (Thbs) matricellular glycoproteins consists of five members (Thbs1-5), and while they are secreted as part of their life-cycle, they also function as intracellular chaperones that mediate the ER stress response, facilitate secretory pathway activity and extracellular matrix production, and facilitate membrane residence of attachment complexes^8–10^. Group A Thbs proteins form trimers that consist of Thbs1 and Thbs2, while group B Thbs proteins form pentamers that consist of Thbs3, Thbs4, and Thbs5^11^. Discrete spatio- and temporal expression patterns characterize Thbs gene expression during development, although in adulthood expression of all 5 genes is typically only observed upon injury^12^. Single *Thbs* gene-deleted mice have been described with a wide variety of phenotypes, although we recently showed that quintuple *Thbs1/2/3/4/5*^*−/−*^ mice are viable as adults^9^, highlighting the fact that these proteins primarily function in stress or injury resolution. In the heart, both Thbs3 and Thbs4 are induced with injury, yet overexpression of Thbs4 is protective while Thbs3 overexpression predisposes to disease with pressure overload stimulation^8,9,13^. Interestingly, Thbs3 uniquely leads to loss of integrin attachment complexes at the sarcolemma in promoting cardiomyopathy while Thbs4 augments membrane content of integrin and dystrophin-glycoprotein attachment complexes as part of its protective profile^8–10^.

Here we investigated the role of Thbs1 and observed that Thbs1 heart-specific transgenic mice have dramatically reduced heart size leading to lethal cardiomyopathy by 16 weeks of age. Mechanistically, Thbs1 but not Thbs3, activate ER-stress via the PERK-eIF2α-ATF4 pathway and prominent autophagic flux in the cardiomyocyte. Exacerbated autophagy, cardiac atrophy, and lethality associated with Thbs1 overexpression was largely rescued by deleting the gene encoding PERK (*Eif2ak3*). Moreover, direct AAV9-mediated overexpression of PERK or ATF4 in the heart drives autophagy and recapitulates the Thbs1-mediated cardiac phenotype. Finally, deletion of Thbs1 in mice protects from cardiac atrophy. Hence, we identify a Thbs1-mediated PERK-eIF2α-ATF4-induced autophagy pathway as a fundamental regulator of protein homeostasis in the stressed heart.

## Results

### Thbs1 is induced in diseased hearts where it mediates ER stress

**Fig. 1:**
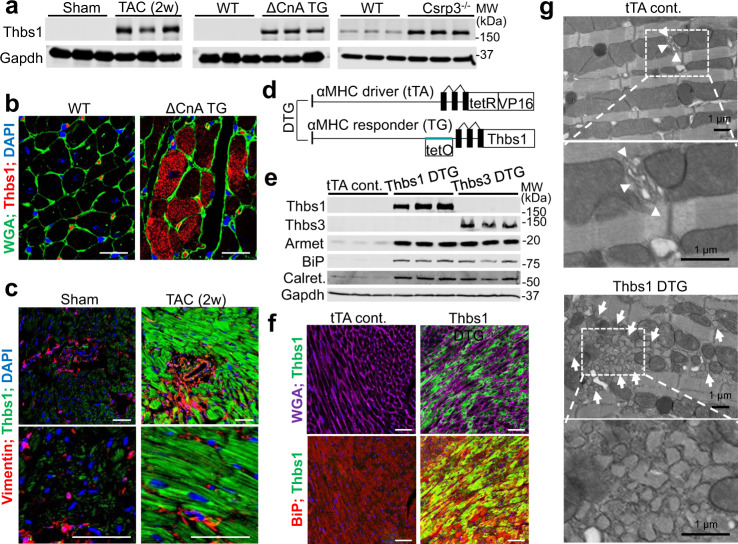
Expression of Thbs1 is induced in diseased hearts where it mediates ER stress. **a** Western blotting for Thbs1 in heart tissue of mice (8–10 weeks of age) subjected to 2 weeks of TAC, that contained the activated calcineurin A transgene (ΔCnA), or are *Csrp3*^*−/−*^, compared to sham-operated or wild-type (WT) control. Gapdh is shown as a processing and loading control. **b** Representative immunohistochemistry for endogenous Thbs1 (red) and cell outlines with wheat germ agglutinin (WGA)-FITC (green) with DAPI-stained nuclei (blue) from hypertrophic ΔCnA transgenic and WT control hearts at 8 weeks of age. Scale bars are 10 μm. **c** Immunohistochemistry for Thbs1 protein (green), vimentin (red) from sham or TAC-operated hearts, 2 weeks later. Scale bars are 50 μm. Nuclei are shown in blue with DAPI. **d** Schematic diagram depicting the inducible double transgenic (DTG) tetracycline-repressor system for inducible overexpression of Thbs1 in the heart. **e** Representative Western blots for Thbs1, Thbs3, Armet, BiP, calreticulin (calret.), and Gapdh as a loading control from hearts of tTA cont., Thbs1 DTG, and Thbs3 DTG mice at 6 weeks of age. **f** Representative immunohistochemistry broken into 2 channels each for overexpressed Thbs1 (green) with WGA-labeled membranes (purple), DAPI for nuclei (blue) and BiP (red) to show ER and the vesicular compartment in Thbs1 DTG hearts at 8 weeks of age. Scale bars are 50 μm. **g** Representative images of transmission electron microscopy of heart sections from tTA cont. and Thbs1 DTG mice at 6 weeks of age. Upper panels: arrowheads indicate ER in tTA cont., white arrows show expanded ER and vesicles only in Thbs1 DTG hearts. Lower panels: enlargement of white dotted boxed area from upper panels. Scale bars are 1 μm. Source data are provided as a Source Data File.

Thbs1 protein expression is strongly induced in the adult mouse heart with injury or stress stimulation, such as during cardiac hypertrophy mediated by 2 weeks of transverse aortic constriction (TAC) or cardiomyocyte-specific overexpression of activated calcineurin (ΔCnA TG)^14^ (Fig. 1a). Thbs1 was also induced in the hearts of *Csrp3*^−/−^ mice^15^, a model of dilated cardiomyopathy (Fig. 1a). Immunohistochemical characterization of hearts from ΔCnA TG mice (Fig. 1b) and mice subjected to 2 weeks of TAC (Fig. 1c) showed that Thbs1 protein was robustly induced and localized inside cardiomyocytes, with appreciably less Thbs1 protein expression observed within fibroblasts or endothelial cells as marked by vimentin expression.

To recapitulate the induction of Thbs1 during heart disease we generated cardiomyocyte-restricted transgenic mice overexpressing Thbs1 using an inducible α-myosin heavy chain (αMHC) promoter system. This inducible tetracycline-regulated double transgenic system^16^ allows for temporal regulation of transgene expression (Fig. 1d). Double transgenic (DTG) mice containing the Thbs1 and the tetracycline transactivator (tTA) transgenes were compared with animals only expressing tTA as controls (tTA cont.). The previously described transgenic mouse line with cardiomyocyte-specific overexpression of Thbs3 was used as a control for ER-stress induction by Thbs proteins in general^9^. Thbs1 DTG hearts showed expression of Thbs1 and an induction of ER resident chaperones Armet, BiP (binding immunoglobulin protein) and calreticulin (Calret.) as observed in Thbs3 DTG hearts (Fig. 1e). Immunohistochemical analysis revealed that Thbs1 predominantly localized inside the cardiomyocyte, where it co-localized with BiP in the ER compartment (Fig. 1f), similar to our previous observations with Thbs3 and Thbs4 overexpression in the heart and skeletal muscle^9,10,13^. Furthermore, ultrastructural analysis by transmission electron microscopy showed ER- and post-ER vesicular expansion in Thbs1 DTG hearts (Fig. 1g, arrows show vesicular expansion). Comparable results were previously described in transgenic animals overexpressing Thbs3 or Thbs4 in the heart and skeletal muscle^9,10,13^, suggesting similar ER residency and induction of the general ER stress response with Thbs1.

### Cardiac Thbs1 expression induces lethal cardiac atrophy

**Fig. 2:**
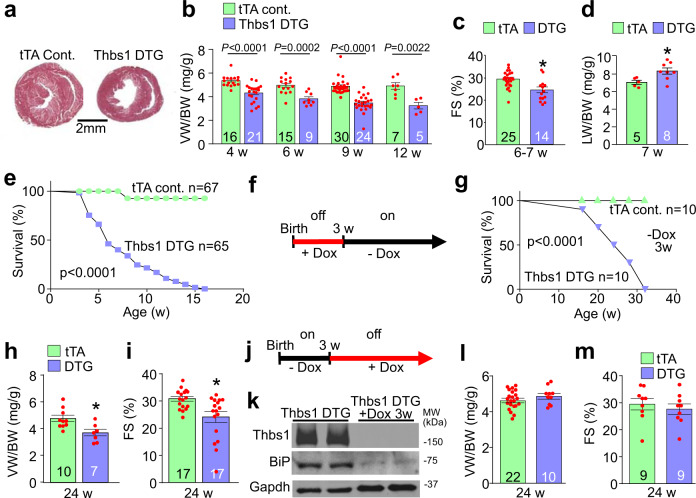
Cardiomyocyte-specific overexpression of Thbs1 induces lethal cardiac atrophy. **a** Low magnification images of tTA cont. and Thbs1 DTG whole mount cardiac histological sections stained with Masson’s trichrome at 6 weeks of age. Scale bar is 2 mm. **b** Ventricular weight-to-body weight (VW/BW) ratio at the indicated time points in tTA cont. and Thbs1 DTG mice. *P*-values are shown in the graph. **c** Echocardiography measured fractional shortening (FS) percentage in the two groups of mice at 6–7 weeks (w) of age. *P = 0.0007 vs tTA cont. **d** Pulmonary edema was analyzed by lung weight-to-body weight (LW/BW) ratios in the indicated groups of mice at 7 weeks of age. *P = 0.0231 vs tTA cont. **e** Kaplan–Meier survival plot of tTA cont. and Thbs1 DTG animals with continuous Thbs1 expression (never Dox; *P* < 0.0001 vs tTA cont.). **f** Experimental regimen of early Dox administration to inhibit Thbs1 transgene expression during embryonic and early postnatal development in Thbs1 DTG animals (Dox was removed at 3w of age). **g** Kaplan–Meier survival plot of tTA cont. and Thbs1 DTG mice treated with Dox as shown in panel “**f**” (*P* < 0.0001 vs tTA cont.). **h** VW/BW ratio and **i** FS percentage at 24 weeks of age given the treatment regimen shown in panel “**f**”. *P = 0.0073 vs tTA cont. for “**h**” and *P = 0.0039 vs tTA cont. for “**i**”. **j** Schematic diagram for administration of Dox beginning at 3 weeks of age to shut down Thbs1 expression thereafter (Dox was given at 3w and beyond). **k** Representative Western blots of protein extracts from hearts of Thbs1 DTG mice at 8 weeks of age in Thbs1 DTG mice as shown in panel “**j**”, assayed for Thbs1, BiP, and Gapdh as a loading control. **l** VW/BW ratio and **m** FS percentage at 24 weeks of age in the mice treated as shown in panel “**j**”. The number of biologically independent animals analyzed is indicated on each graph. Statistical analysis was performed using two-tailed Student’s *t* test (**b**–**d**, **h**, **i**, **l**, **m**), or survival analysis by two-tailed log-rank test (**e**, **g**). Error bars are ±standard error of the mean. Source data are provided as a Source Data File.

Overexpression of Thbs3 or Thbs4 in the heart or skeletal muscle had no effect at baseline, and Thbs4 DTG hearts were even protected from decompensation induced by pressure overload or myocardial infarction injury^9,10,13^. However, cardiomyocyte-specific overexpression of Thbs1 showed a profound and progressive reduction in ventricular weight-to-body weight ratio (VW/BW) (Fig. 2a, b) starting at 4 weeks of age. Compared to tTA controls, Thbs1 DTG mice also displayed a significant reduction in fractional shortening and increased incidence of pulmonary edema at 6–7 weeks of age (Fig. 2c, d). Thbs1 overexpressing transgenic mice had reduced survival with no animals living past 16 weeks of age (Fig. 2e). Importantly, using doxycycline (Dox) to repress transgene expression and bypass effects during early postnatal development, we showed that adult-specific induction of Thbs1 expression in the heart still led to lethal cardiomyopathy, as none survived past 32 weeks of age (Fig. 2f, g). Analysis of these Thbs1 DTG (-Dox 3w) animals again revealed reduced VW/BW ratio (Fig. 2h) and diminished fractional shortening at 24 weeks of age (Fig. 2i).

We also examined mice in which the Thbs1 transgene was only active for the first 3 weeks of postnatal development, after which Dox was given (+Dox 3w) to continuously repress expression (Fig. 2j, k). With repression there was also reduced induction of BiP protein within the ER (Fig. 2k). Importantly, VW/BW ratio (Fig. 2l) and heart function (Fig. 2m) at 24 weeks of age were not affected by this early window of cardiac Thbs1 overexpression, indicating that Thbs1 did not inherently change cardiac cellularity or remodeling, but instead appeared to only affect growth/atrophy dynamics in real time.

### Thbs1 in heart is independent of TGFβ and endothelial cells

**Fig. 3:**
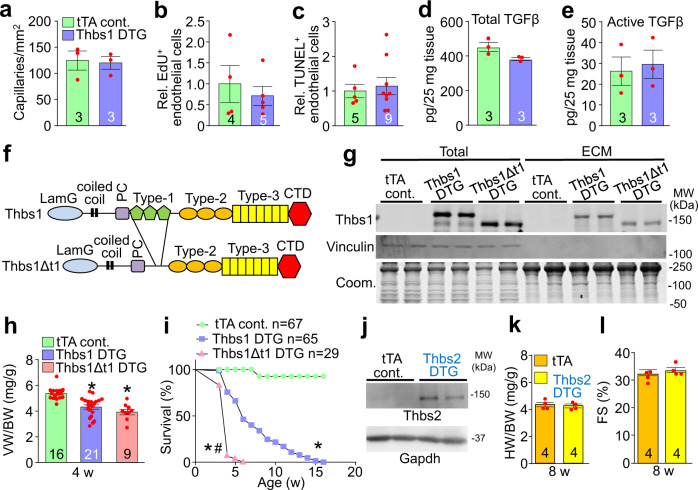
Endothelial cells and TGFβ are not affected by cardiac Thbs1 overexpression. **a** Quantification of capillary number per mm^2^ of tissue from histological sections of tTA cont. and Thbs1 DTG hearts stained with isolectin B4 at 6 weeks of age. **b** Quantification of endothelial cell proliferation as measured by EdU incorporation co-labeled with CD31 in tTA cont. and Thbs1 DTG hearts at 6 weeks of age. **c** Quantification of endothelial cell apoptosis detected by TUNEL staining co-labeled with isolectin B4 in tTA cont. and Thbs1 DTG hearts at 6 weeks of age. **d** ELISA-based quantification of total TGFβ and **e** active TGFβ in protein extracts from tTA cont. or Thbs1 DTG hearts at 6 weeks of age. **f** Schematic diagram of WT Thbs1 domain structure and the Thbs1Δt1 mutant lacking the Thbs1 type-1 repeat domain region. **g** Representative western blot analysis for Thbs1 from total protein extracts (Total) and extracellular matrix (ECM) extracts from hearts of tTA cont., Thbs1 DTG, and Thbs1 DTG Δt1 mice at 4 weeks of age. Vinculin is presented as cytosolic control. Coomassie stained (Coom.) gel is shown as loading control. **h** VW/BW ratio at 4 weeks of age in the indicated groups of mice. *P < 0.0001 versus tTA cont.; statistical analysis was performed using one-way ANOVA and Tukey multiple comparisons test. **i** Kaplan–Meier survival plot of tTA cont., Thbs1 DTG, and Thbs1Δt1 DTG animals. *P < 0.0001 vs tTA cont. #*P* < 0.0001 vs Thbs1 DTG; both analyzed by two-tailed log-rank test. The same data from Fig. 2e are shown again here for tTA cont. and Thbs1 DTG mice (same strain and ages and sex ratio mix). **j** Representative western blots for Thbs2 and Gapdh as loading control, from heart protein extracts from tTA cont. and Thbs2 DTG mice at 8 weeks of age. **k** Heart weight (HW)/BW ratio, and **l** FS percentage at 8 weeks of age from tTA cont. and Thbs2 DTG mice. The number of biologically independent animals analyzed is indicated on each graph. Error bars are ±standard error of the mean. Source data are provided as a Source Data File.

Thbs1 release into the extracellular space can modify endothelial cell function by inhibiting proliferation or inducing cell death^17^. However, overexpression of Thbs1 in the heart had no effect on capillary number (Fig. 3a), endothelial cell proliferation as measured by 5-ethynyl-2´-deoxyuridine (EdU) incorporation (Fig. 3b) or apoptosis detected by terminal deoxynucleotidyl transferase dUTP nick end labeling (TUNEL) staining (Fig. 3c). Thbs1 has also been described as an endogenous activator of transforming growth factor-β (TGFβ)^18^. However, analysis of total and active levels of TGFβ by ELISA from heart tissue lysates showed no differences between Thbs1 DTG and tTA control mice (Fig. 3d, e).

The Thbs1 type-1 repeat domains are implicated in mediating inhibition of endothelial cell activity and in the direct binding and activation of TGFβ in vivo^18,19^. To analyze these potential effector functions in directing the atrophy phenotype, we generated a second Thbs1 overexpressing transgenic mouse line devoid of the Thbs1 type-1 repeat region (Thbs1Δt1 DTG) (Fig. 3f). Protein analysis revealed that both full-length Thbs1 and mutant Thbs1Δt1 are expressed and secreted in the extracellular matrix with the same efficiency (Fig. 3g). Importantly, cardiomyocyte-restricted overexpression of the Thbs1Δt1 showed a similar loss of heart mass and a significantly enhanced lethal phenotype compared to overexpression of full-length Thbs1 DTG, even though levels of the Thbs1Δt1 protein were ~50% lower compared with WT Thbs1 (Fig. 3h, i). Finally, we also generated cardiac-specific Thbs2 overexpressing DTG mice (Fig. 3j–l). This is mechanistically important because Thbs2 is in the same trimeric group A class as Thbs1, and both have the type-1 repeat domain^11^. However, Thbs2 overexpression (Fig. 3j) did not cause cardiac atrophy or cardiac dysfunction (Fig. 3k, l). Collectively, these results indicate that the lethal and cardiac atrophic phenotype observed with Thbs1 overexpression is not due to direct effects on cardiac endothelial cells or aberrant TGFβ activation in the heart.

CD47 and CD36 cell surface proteins were originally proposed to serve as Thbs1 receptors, where they mediate signaling associated with secreted extracellular Thbs1^11^. While the field now understands that CD36 and CD47 are multiligand receptors with additional important functions in fatty acid transport and immune tolerance, respectively, we examined if these membrane proteins affected Thbs1-mediated cardiac atrophy. Hence, we crossed Thbs1 DTG mice with either *Cd47*^*−/−*^ or *Cd36*^*−/−*^ mice. Heart tissue extracts of Thbs1 DTG crossed with *Cd47*^*−/−*^ mice showed complete absence of CD47 protein and slightly increased levels of Thbs1 in the heart compared to Thbs1 DTG hearts (Supplementary Fig. 1a). However, loss of *Cd47* did not affect the Thbs1-mediated cardiac atrophy (Supplementary Fig. 1b), nor did it prolong survival of Thbs1 DTG *Cd47*^*−/−*^ animals (Supplementary Fig. 1c). Deletion of *Cd36*^*−/−*^ in the Thbs1 DTG background was confirmed by Western blot analysis (Supplementary Fig. 1d), which also had no effect on Thbs1-induced atrophy nor did it rescue the observed premature lethality (Supplementary Fig. 1e, f). Taken together, these results indicate that Thbs1-mediated cardiac atrophy and premature lethality are independent of CD36 or CD47.

Thbs3 and Thbs4 mediate ER stress and ER vesicular expansion through the transcription factor ATF6α^20^, and Thbs1 overexpression similarly induced ER stress and ER vesicular expansion (Fig. 1e, g). Here we crossed Thbs1 DTG mice into the *Atf6*^*−/−*^ genetic background, which did not reduce the atrophy associated with the Thbs1 DTG, nor did it extend viability (Supplementary Fig. 1g, h). In fact, loss of ATF6α, as well as loss of CD36, significantly enhanced the cardiomyopathic lethal phenotype associated with Thbs1 overexpression in the heart (Supplementary Fig. 1f, h).

### Thbs1 binds and activates a PERK-mediated ER-stress response

**Fig. 4:**
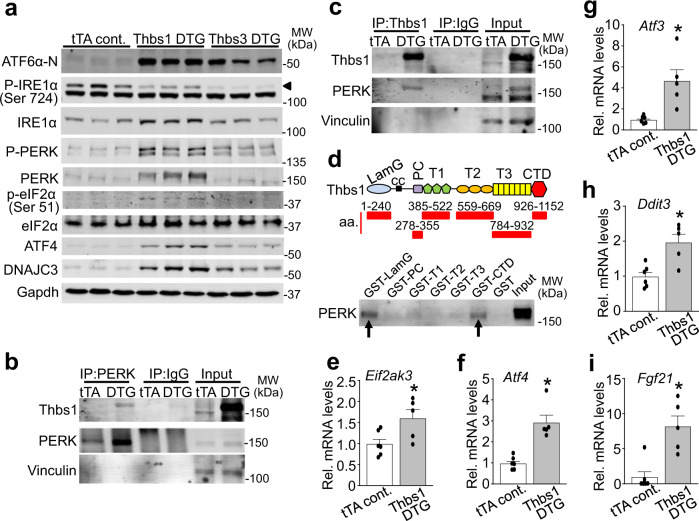
Thbs1 binds and activates a PERK-mediated ER stress response. **a** Representative western blots for ATF6α-N (50 kDa, nuclear), phospho-IRE1α (Ser 724; arrowhead), total IRE1α, phospho- and total PERK, phospho-eIF2α (Ser 51), total-eIF2α, ATF4, and DNAJC3. Phospho-PERK was analyzed using Phos-tag gels. The heart protein extracts are from the indicated mice at 4 weeks of age. Gapdh serves as a processing and loading control. **b**, **c** Western blot for Thbs1 and PERK following immunoprecipitation (IP) of PERK (**b**) or Thbs1 (**c**) from protein extracts of tTA cont. and Thbs1 DTG hearts at 6 weeks of age. IPs with corresponding IgG served as negative controls. Vinculin is an input loading control. **d** Schematic diagram of Thbs1 with the GST-Thbs1 fusion proteins regions shown (red bars). Below the schematic a representative western blot for PERK following GST pull-down with the different Thbs1 domains from primary neonatal rat ventricular cardiomyocyte extracts. GST protein serves as a negative control. **e**-**i**, Quantitative RT-PCR for *Eif2ak3* (PERK protein; *P = 0.0317 vs tTA control), *Atf4* (*P = 0.0006 vs tTA control), *Atf3* (*P = 0.0058 vs tTA control), *Ddit3* (Chop protein; *P < 0.0001 vs tTA control)) and *Fgf21* (*P = 0.0023 vs tTA control) from mRNA isolated from hearts of tTA cont. (n = 6 biologically independent animals) and Thbs1 DTG mice (n = 5 biologically independent animals) at 6 weeks of age. Statistical analysis was performed using two-tailed Student’s *t* test. Error bars are ±standard error of the mean. Source data are provided as a Source Data File.

Overexpression of Thbs1, 3, and 4 in heart or skeletal muscle^8–10,13^ each induced a similar ER stress profile (Fig. 1e). However, Thbs1 was unique in that it alone caused lethal cardiomyopathy leading us to investigate more deeply into the ER stress response in the Thbs1 and Thbs3 transgenic hearts (Fig. 4a). Indeed, while both the Thbs1 and Thbs3 transgenic hearts displayed enhanced levels of nuclear ATF6α (ATF6α-N) and repressed IRE1α phosphorylation, Thbs1 strongly activated and induced the PERK-ATF4 ER stress effectors (Fig. 4a). Importantly, eIF2α phosphorylation was uniquely enhanced with Thbs1 overexpression, while ATF4 and its direct transcriptional target DNAJC3 were most highly induced in Thbs1 DTG hearts compared with tTA control or Thbs3 DTG hearts (Fig. 4a).

Previous studies have shown that Thbs proteins can interact with ER resident effectors while maturing in the secretory pathway, including BiP and ATF6α^8,9,13,21^. In line with these findings, immunoprecipitation (IP) experiments from protein extracts showed that Thbs1 and PERK interact in Thbs1 DTG hearts, but not in tTA control hearts or when performing an IgG control IP (Fig. 4b, c). Furthermore, we also investigated the structural domains of Thbs1 that interact with PERK using glutathione S-transferase (GST) fusion fragments^22^. Interestingly, this identified both the N-terminal laminin G (LamG) domain and the carboxy-terminal domain (CTD) of Thbs1 as PERK interacting in protein extracts of primary neonatal rat ventricular cardiomyocytes (NRVMs; Fig. 4d). Nevertheless, future confirmation of this finding is warranted as these GST fusion fragments are derived from a bacterial expression system where mammalian glycosylation is absent, and chaperones needed to mediate proper disulfide bond formation are lacking. Finally, quantitative PCR analysis in Thbs1 DTG hearts revealed upregulation of *Eif2ak3* (PERK) at the mRNA level (Fig.4e), suggesting a transcriptional feed forward mechanism. *Atf4* and known downstream targets such as *Atf3, Ddit3* (Chop), and fibroblast growth factor 21 (*Fgf21*) were also upregulated in Thbs1 DTG hearts compared with controls (Fig. 4f–i). Such results are interesting given that ATF4 and its downstream effector Fgf21 mediate atrophic remodeling in skeletal muscle^23,24^. Taken together, our results suggest a mechanism whereby Thbs1 induces lethal cardiac atrophy, in part, through the PERK-eIF2α-ATF4 ER stress pathway.

### Thbs1 induces autophagy and lysosomal protein degradation

**Fig. 5:**
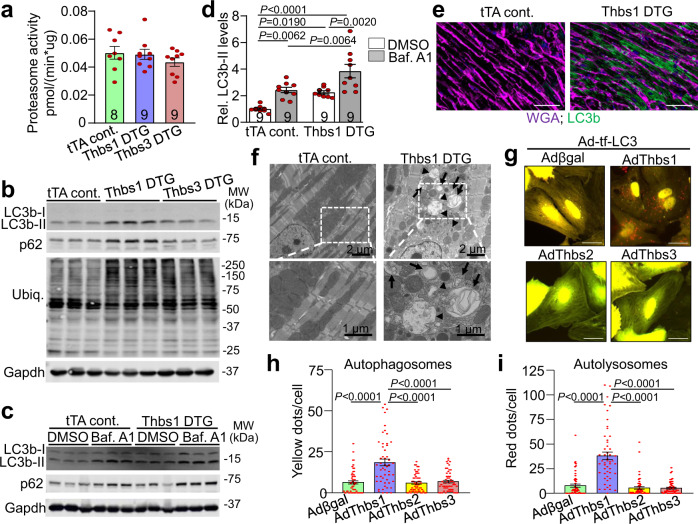
Thbs1 induces autophagy and lysosomal protein degradation. **a** 20S chymotrypsin-like proteasome activity measured in heart tissue of tTA cont., Thbs1 DTG, and Thbs3 DTG mice at 4 weeks of age. **b** Representative western blots for LC3b, p62, ubiquitin-conjugated proteins (Ubiq.), and Gapdh as loading control from hearts of tTA cont., Thbs1 DTG, and Thbs3 DTG mice at 4 weeks of age. **c** Representative western blots for LC3b, p62, and Gapdh as loading control from hearts of tTA cont., Thbs1 DTG treated with dimethyl sulfoxide (DMSO) as vehicle or bafilomycin A1 (Baf. A1) to inhibit autolysosome degradation at 6 weeks of age. **d** Quantitative analysis of LC3b-II protein levels relative to Gapdh from the experiment shown in “**c**”. Analysis of p62 protein levels is shown in Supplementary Fig. 3c. Data are represented as fold change compared to tTA cont. with DMSO. **e** Representative immunohistochemistry for LC3b protein (green) and WGA (purple) from heart sections of tTA cont. and Thbs1 DTG mice at 8 weeks of age. Scale bars are 50 μm. **f** Representative transmission electron microscopy of Thbs1 DTG and tTA cont. heart sections at 6 weeks of age. Autophagosomes (arrow) appear as double-membrane vesicles while autolysosomes are single membrane structures (arrowheads). Lower panels are enlargements of white dotted boxed area from upper panels. Scale bars are 2 μm and 1 μm, respectively. **g**–**i** Representative micrographs of fluorescent LC3 puncta (**g**) and quantification thereof (**h**, **i**) in cultured primary neonatal rat ventricular myocytes 48 h after infection with indicated adenoviruses to overexpress tandem mRFP-GFP-LC3 (Ad-tf-LC3) indicator with either Thbs1 (*n* = 51), Thbs2 (*n* = 57), Thbs3 (*n* = 57), or βgal control (*n* = 53). ‘*N*’ represents biologically independent cells; yellow dots represent autophagosomes, whereas free red dots indicated autolysosomes. Scale bars are 50 μm. The number of biologically independent animals analyzed for “**a**” and “**d**” is indicated on each graph. All statistical analysis were performed using one-way ANOVA and Tukey multiple comparisons test; error bars are ±standard error of the mean; *P*-values for panel **d**, **h**, **i** are shown in the graph. Source data are provided as a Source Data File.

We next investigated the molecular basis for Thbs1-mediated cardiac atrophy. The ubiquitin-proteasome system (UPS) and autophagy are two major mechanisms responsible for protein degradation in the heart^2^. However, no significant change in 20S proteasome activity was observed in Thbs1 transgenic hearts compared to Thbs3 DTG and tTA controls (Fig. 5a). *Fbxo32* (Atrogin-1) and *Trim63* (MuRF1) transcript levels were also unaffected by Thbs1 overexpression (Supplementary Fig. 2a, b). Interestingly, levels of certain ubiquitinated proteins and several key components of the ER-associated degradation (ERAD) machinery in the heart, including valosin-containing protein (VCP)/p97, Homocysteine Inducible ER Protein With Ubiquitin Like Domain 1 (Herpud1) and sel-1 suppressor of lin-12-like 1 (Sel1L), but not derlin-1 (Derl1) and ER degradation-enhancing alpha-mannosidase-like protein 1 (Edem1) were increased in the Thbs1 transgenic hearts, compared to tTA control (Fig. 5b and Supplementary Fig. 2c, d). Nevertheless, similar changes were observed in the Thbs3 transgenic hearts, which do not develop a lethal cardiac atrophy (Fig. 5b and Supplementary Fig. 2c, d).

Emerging data suggest a regulatory role for the PERK-eIF2α-ATF4 ER stress axis in autophagy^6,7^. Similarly, markers of autophagy, microtubule-associated proteins 1A/1B light chain 3B-II (LC3b-II) and p62 were uniquely increased in Thbs1 DTG hearts compared with tTA control or Thbs3 DTG hearts (Fig. 5b and Supplementary Fig. 3a, b). Thbs1 DTG animals and tTA controls were treated with the inhibitor of autolysosomal degradation bafilomycin A1 (Baf. A1) to more closely investigate autophagic flux in the heart. A significant increase in LC3b-II and p62 was observed at baseline in Thbs1 DTG hearts that was further enhanced in Baf. A1 treated animals, thus confirming enhanced autophagic flux (Fig. 5c, d and Supplementary Fig. 3c). In agreement, immunohistochemical analysis using a LC3b specific antibody revealed strong vesicular staining only in Thbs1 DTG heart histological sections compared with tTA control; this was confirmed at the ultrastructural level by electron microscopy (Fig. 5e, f and Supplementary Fig. 3d, e).

To further demonstrate that Thbs1 enhances autophagic flux in cardiomyocytes, cultured primary neonatal rat ventricular cardiomyocytes (NRVMs) were infected with adenoviruses to overexpress tandem fluorescent monomeric red fluorescent protein (mRFP)- green fluorescent protein (GFP)-LC3 (tf-LC3)^25^ together with Thbs1, Thbs2, Thbs3, or β-galactosidase (βgal) control. After incorporation into autophagosomes, the tf-LC3 can effectively differentiate autophagosomes from autolysosomes because the acidic environment in autolysosomes quenches GFP but not mRFP fluorescence. Consistent with our in vivo results, we observed that Thbs1, but not Thbs2, Thbs3, or βgal significantly enhanced the number of autophagosomes (yellow puncta) and autolysosomes (red puncta), confirming increased autophagic flux in NRVMs upon Thbs1 overexpression (Fig. 5g–i and Supplementary Fig. 3f–h).

Finally, previous studies have also associated the PERK-eIF2α-ATF4 ER stress axis with increased expression of tuberous sclerosis protein 2 (TSC2) activator, and thus mTOR inhibitor DNA damage inducible transcript 4 (DDIT4)^26^. Activation of AKT/mTOR pathway promotes protein synthesis and cellular growth, whereas its inactivation leads to atrophic remodeling^27^. However, investigation of AKT, mTOR, and downstream targets such as ribosomal protein S6 kinase (p70S6K) revealed no overt changes in total protein levels in the hearts of Thbs1 DTG mice, although AKT phosphorylation was reduced compared with tTA control or Thbs3 overexpressing mice (Supplementary Fig. 3i).

Collectively, these results suggest a mechanism whereby Thbs1 mediates lethal cardiomyopathy through PERK-eIF2α-ATF4-induced and enhanced autophagic protein degradation in the heart, like previous results observed with Forkhead box O3 (FOXO3) mediated overactivation of autophagy in the heart^28^.

### *Thbs1*^*−/−*^ mice have greater hypertrophy and less atrophy

**Fig. 6:**
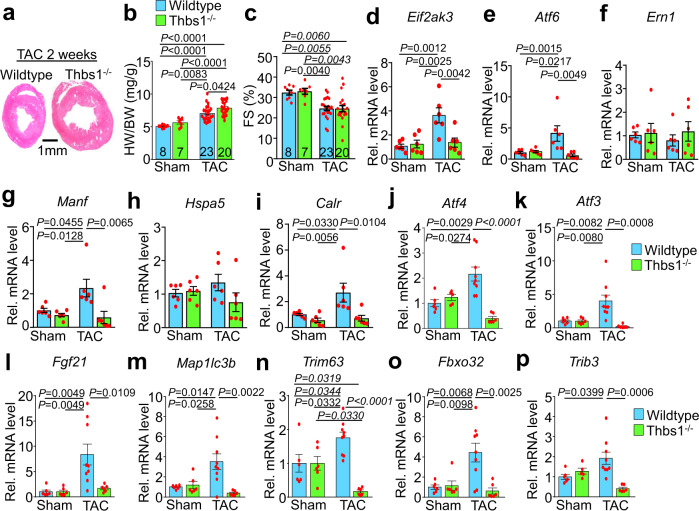
Loss of *Thbs1* augments hypertrophic growth and restricts atrophy in the heart. **a** Low magnification images of wild type and *Thbs1*^*−/−*^ whole mount cardiac histological sections stained with Hematoxylin & Eosin, 2 weeks after TAC surgery at 8 weeks of age. Scale bar is 1 mm. **b** HW/BW ratio and **c** FS percentage 2 weeks after TAC or sham surgery at 8 weeks of age. The number of biologically independent animals analyzed is indicated on the graphs for panels “**b**–**d**”. **d**–**p** Quantitative RT-PCR results for *Eif2ak3* (PERK protein)*, Atf6, Ern1* (IRE1α protein)*, Manf* (Armet protein)*, Hspa5* (BiP protein)*, Calr* (Calreticulin protein), *Atf4, Atf3, Fgf21, Map1lc3b* (LC3b protein)*, Trim63* (MuRF1 protein)*, Fbxo32* (Atrogin-1 protein), and *Trib3* mRNA isolated from hearts of wild type and *Thbs1*^*−/−*^ mice, 2 weeks after TAC or sham surgery at 8 weeks of age. For panels “**d**–**i**”, *n* = 6 biologically independent samples per group; for panels “**j** and **p**”, *n* = 6 biologically independent samples for sham wild type and *Thbs1*^*−/−*^ 2 weeks after TAC surgery, *n* = 5 biologically independent samples for sham *Thbs1*^*−/−*^ and *n* = 9 biologically independent samples for wild-type 2 weeks after TAC surgery; for panels “**k**–**o**”, *n* = 6 biologically independent samples for sham wild-type, sham *Thbs1*^*−/−*^ and *Thbs1*^*−/−*^ 2 weeks after TAC surgery and *n* = 9 biologically independent samples for wild-type 2 weeks after TAC surgery. Data are represented as fold expression over sham wild type. All statistical analysis was performed using one-way ANOVA and Tukey multiple comparisons test. *P*-values are shown in each graph. Error bars are ±standard error of the mean. Source data are provided as a Source Data File.

Increased levels of Thbs1 protein were detected after TAC mediated cardiac injury (Fig. 1a). Moreover, western blot analysis revealed that Thbs1 overexpression in the heart induced a profile of ER stress markers like those observed during cardiac hypertrophy mediated by 2 weeks of TAC, including changes in nuclear ATF6α, phosphorylated and total PERK, phosphorylated and total IRE1α, ATF4 and calreticulin, and to a lesser extent Armet and BiP (Supplementary Fig. 4). Previous studies analyzing *Thbs1*^*−/−*^ animals revealed that these mice have increased hypertrophic growth upon pressure overload^29^. Similarly, here we observed that *Thbs1*^*−/−*^ mice responded with significantly increased heart weight to body weight (HW/BW) ratios 2 weeks after TAC surgery compared with wild-type (WT) controls, but without a further effect on function (Fig. 6a–c). This result suggests that induction of endogenous Thbs1 after TAC stimulation normally mediates an anti-hypertrophic effect, presumably through greater autophagy. To further characterize the effect of Thbs1 on protein homeostasis in the heart we utilized a 48-h fasting model. When WT animals were fasted for 48 h, Thbs1 mRNA levels were found to be significantly increased (Supplementary Fig. 5a). Remarkably, *Thbs1*^*−/−*^ mice showed significantly less loss of heart weight and body weight with 48 h of fasting compared with matched controls (Supplementary Fig. 5b, c), which again suggests that induction of Thbs1 is likely involved in cardiac atrophy.

Quantitative PCR analysis of ER stress mediators revealed a general profile with increased mRNA levels of *Eif2ak3* (PERK) and *Atf6*, and downstream target genes *Manf* (Armet) and *Calr* (calreticulin) in WT hearts in response to 2 weeks of TAC surgery, whereas no changes were observed for *Ern1* (IRE1α) and *Hspa5* (BiP; Fig. 6d–i). Furthermore, the TAC-induced expression of *Eif2ak3, Atf6*, *Manf*, and *Calr* was blunted in *Thbs1*^*−/−*^ hearts (Fig. 6d–i). More importantly and in line with our previous findings, induction of hypertrophy by TAC and atrophy by fasting resulted in similar transcriptional changes associated with the PERK/ATF4/autophagy pathway. Specifically, quantitative mRNA PCR analysis revealed upregulation of *Atf4* and its downstream target genes *Atf3*, *Fgf21*, *Map1lc3b* (LC3b), and *Trim63* (MuRF1) in WT animals in both models, which was abrogated in *Thbs1*^*−/−*^ hearts (Fig. 6j–n and Supplementary Fig. 5d–h). *Fbxo32* (Atrogin-1) was upregulated in both models in WT animals, however, this effect was only blunted in *Thbs1*^*−/−*^ hearts that underwent TAC surgery and not the fasting regimen (Fig. 6o and Supplementary Fig. 5i). A similar effect was observed analyzing *Trib3*, which was upregulated in WT animals after TAC surgery and downregulated in *Thbs1*^*−/−*^ hearts, but unaffected in fasted animals of either genotype (Fig. 6p and Supplementary Fig. 5j). These results suggest a role for Thbs1 in regulating ATF4-dependent gene expression changes that underlie atrophic remodeling.

### Thbs1 induces PERK/ATF4 to facilitate cardiac atrophy

**Fig. 7:**
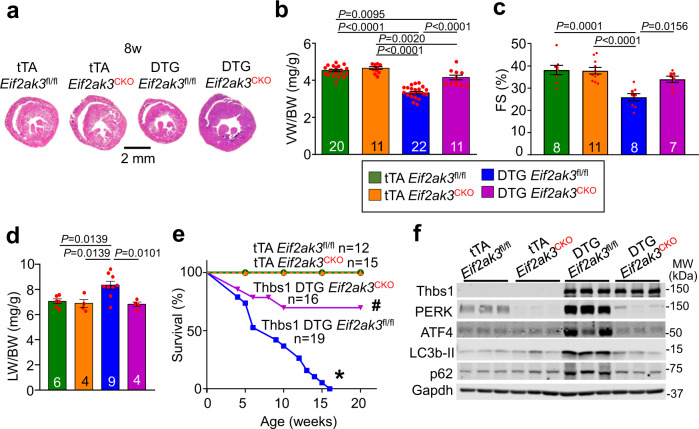
Thbs1 induces PERK/ATF4 to facilitate cardiac atrophy. **a** Low magnification cardiac histological images from tTA cont. *Eif2ak3*^*fl/fl*^ (PERK), tTA *Eif2ak3*^*fl/fl*^ βMHC-Cre (*Eif2ak3*^*CKO*^), Thbs1 DTG *Eif2ak3* ^*fl/fl*^, and Thbs1 DTG *Eif2ak3*^*CKO*^ mice stained with Masson’s trichrome at 8 weeks of age. Scale bar is 2 mm. **b** VW/BW ratio, **c** FS percentage and **d** LW/BW ratio at 8 weeks of age in the indicated groups of mice. The number of biologically independent animals analyzed and *P*-values are indicated on the graphs for panels “**b**–**d**”. Statistical analysis was performed using one-way ANOVA and Tukey multiple comparisons test for panels “**b**–**d**”. Error bars are ±standard error of the mean. **e** Kaplan–Meier survival plot from tTA cont. *Eif2ak3*^*fl/fl*^, Thbs1 DTG *Eif2ak3*^*fl/fl*^, tTA cont. *Eif2ak3*^*CKO*^ and Thbs1 DTG *Eif2ak3*^*CKO*^. The number of biologically independent animals analyzed are indicated on the graph. Statistical analysis was performed using a two-tailed log-rank test. *P < 0.0001 vs tTA cont. *Eif2ak3*^*fl/fl*^, *Eif2ak3*^*CKO*^ and Thbs1 DTG *Eif2ak3*^*CKO*^, #*P* = 0.0678 vs tTA cont. *Eif2ak3*^*fl/fl*^, and #*P* = 0.0404 vs *Eif2ak3*^*CKO*^. **f** Representative western blots for Thbs1, PERK, ATF4, LC3b, and p62 from cardiac protein extracts isolated from the groups shown at 8 weeks of age. Protein extracts were from dissociated adult mouse heart cardiomyocytes. Gapdh serves as a loading control. Source data are provided as a Source Data File.

The unique induction of PERK/ATF4 and enhanced autophagic flux in Thbs1 DTG hearts versus Thbs3 overexpression suggested a potential mechanistic basis for how Thbs1 functions in the heart to promote atrophy upon injury or stress stimulation. To directly examine this, we used *Eif2ak3*-loxP-targeted (*Eif2ak3*^*fl/fl*^) mice crossed with β-myosin heavy chain (βMHC) promoter driven Cre transgenic animals to establish cardiomyocyte-specific deletion of the *Eif2ak3* gene (PERK protein) in the heart (*Eif2ak3*^*CKO*^) with Thbs1 overexpression (DTG *Eif2ak3*^*CKO*^). Deletion of *Eif2ak3* in the heart significantly antagonized cardiac atrophy, dysfunction and pulmonary edema due to Thbs1 overexpression at 8 weeks of age (Fig. 7a–d), and the lethal cardiomyopathic phenotype was largely rescued (Fig. 7e). Protein analysis by western blot revealed near absent protein levels of PERK in the DTG *Eif2ak3*^*CKO*^ hearts compared to DTG *Eif2ak3* controls (Fig. 7f), and induction of ATF4, LC3b-II and p62 due to Thbs1 overexpression were all normalized upon deletion of *Eif2ak3* in the heart (Fig. 7f and Supplementary Fig. 6a–c). Taken together, these data provide genetic evidence that Thbs1 induction mediates cardiac atrophy through PERK/ATF4 signaling in the heart.

### Overexpression of PERK mediates cardiac atrophy

**Fig. 8:**
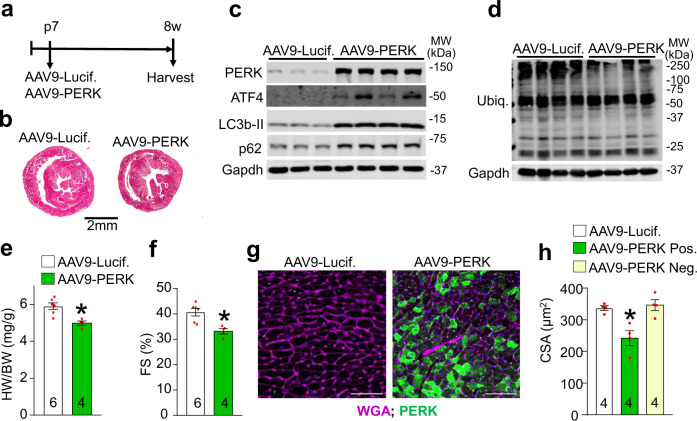
Overexpression of PERK mediates cardiac atrophy. **a** Schematic diagram depicting the experimental protocol. Either 1E11 genomic copies of adeno-associated virus 9 (AAV9)-PERK or AAV9-luciferase (Lucif.) control were injected into the mediastinum of 7-day-old WT mouse pups. Hearts were harvested at 8 weeks of age for further analysis. **b** Low magnification of whole mount cardiac histological images from mice injected with AAV9-PERK or AAV9-Lucif. control stained with Masson’s trichrome at 8 weeks of age. Scale bar is 2 mm. **c** Representative western blots for PERK, ATF4, LC3b, and p62 from cardiac protein extracts of 8-week-old mice injected with either AAV9-Lucif. or AAV9-PERK. Gapdh serves as a loading control. **d** Representative western blot for ubiquitin-conjugated proteins (Ubiq.) and Gapdh as loading control on cardiac protein extracts of 8-week-old mice injected with either AAV9-Lucif. or AAV9-PERK. **e** HW/BW ratio (*P = 0.0051 vs AAV9-Lucif.) and **f** FS% at 8 weeks of age in the 2 indicated groups of mice. *P = 0.0060 vs AAV9-Lucif. **g** Representative immunohistochemistry for PERK (green), nuclei with DAPI (blue) and WGA (purple)-stained membranes from heart sections of AAV9-PERK or AAV9-Lucif. injected mice killed at 8 weeks of age. Scale bars are 100 μm. **h** Quantitative analysis of AAV9-Lucif. versus or AAV9-PERK positive (Pos.) and negative (Neg.) cross sectional area (CSA) determined by WGA staining of cardiac histological sections. *P = 0.0087 vs AAV9-Lucif. and *P = 0.0050 vs AAV9-PERK Neg. The number of biologically independent animals analyzed are indicated on the graphs. Statistical analysis was performed using a two-tailed Student’s *t*-test in panels “**e**, **f**”, and one-way ANOVA and Tukey multiple comparisons test in panel “**h**”. All error bars are ±standard error of the mean. Source data are provided as a Source Data File.

To recapitulate the induction of PERK in the adult myocardium by Thbs1, we utilized an adeno-associated virus serotype-9 (AAV9)-mediated approach to overexpress either PERK or luciferase (control) in the hearts of wild-type mice injected at postnatal (p) day 7 (Fig. 8a, b). Seven weeks later, AAV9-mediated overexpression of PERK resulted in activation of ATF4, LC3b-II, and p62 as shown by western blot analysis, whereas no changes were observed in the levels of ubiquitinated proteins as compared to AAV9-luciferase injected control littermates (Fig. 8c, d and Supplementary Fig. 7a–e). In addition, overexpression of PERK did not appear to impact the levels of general ER stress markers Armet, BiP, and calreticulin, nor did it alter the protein levels of Thbs1 compared to AAV9-Luciferase injected controls (Supplementary Fig. 7f). However, AAV9-mediated overexpression of PERK resulted in atrophic remodeling of the heart as shown by reduced heart size, HW/BW ratio, and cardiac function at 8 weeks of age (Fig. 8b, e, f). Furthermore, enhanced levels of PERK reduced cardiomyocyte surface area of infected, PERK positive cardiomyocytes (AAV9-PERK Pos.) but not neighboring uninfected cardiomyocytes (AAV9-PERK Neg.), as determined by PERK immunohistochemistry (Fig. 8g, h). Collectively, these data indicate a clear mechanism whereby PERK is sufficient to induce autophagic remodeling in the heart.

### ATF4 is sufficient to induce cardiac atrophy

**Fig. 9:**
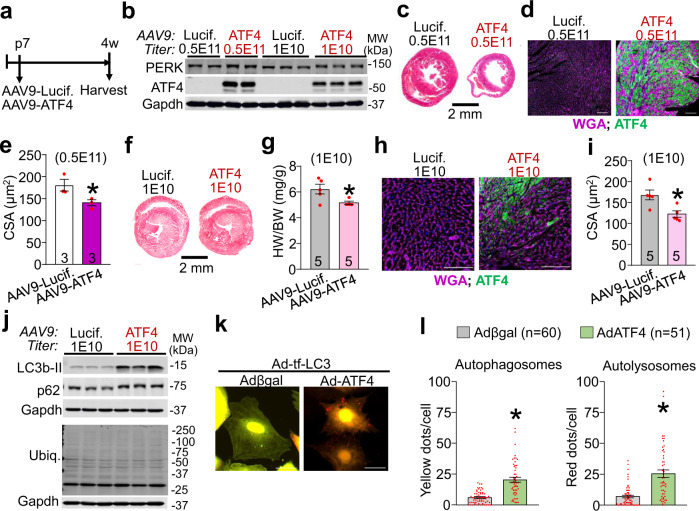
ATF4 is sufficient to induce cardiac atrophy. **a** Schematic diagram depicting the experimental protocol. Either 0.5E11 or 1E10 genomic copies (gc) of AAV9-ATF4 or AAV9-Lucif. control were injected into the mediastinum of 7-day-old wild-type mouse pups. Hearts were harvested at 4 weeks of age for further analysis. **b** Representative western blots for PERK and ATF4 from cardiac protein extracts of 4-week-old mice treated with the indicated AAV9. Gapdh serves as a loading control. **c**, **d** Representative heart sections with Masson’s Trichrome staining (**c**) and immunohistochemistry for ATF4 (green) and WGA (purple)-stained membranes and nuclei with DAPI (blue) (**d**) of AAV9-ATF4 or AAV9-Lucif. injected mice (both 0.5E11 gc) at 4 weeks of age. Scale bars are 2 mm and 100 μm, respectively. **e** CSA of AAV9-Lucif. versus AAV9-ATF4 positive cardiomyocytes determined by ATF4 and WGA staining of histological sections as shown in panel “**d**”. *P = 0.0548 vs AAV9-Lucif. **f** Representative Masson’s trichrome stained images of hearts from mice injected with 1E10 gc AAV9-Lucif or -ATF4 and harvested at 4 weeks of age. Scale bar is 2 mm. **g** HW/BW ratio at 4 weeks of age in the indicated groups of mice. *P = 0.0235 vs AAV9-Lucif. **h** Representative immunohistochemistry for ATF4 (green), nuclei with DAPI (blue) and WGA (purple)-stained membranes from heart sections of AAV9-ATF4 or AAV9-Lucif. injected mice (both 1E10 gc) at 4 weeks of age. Scale bars are 100 μm. **i** CSA of ATF4 positive cardiomyocytes determined by ATF4 and WGA staining as shown in panel “**h**”. *P = 0.0116 vs AAV9-Lucif. **j** Representative western blots for LC3b, p62, and ubiquitin-conjugated proteins (Ubiq.) from cardiac protein extracts of 4-week-old mice treated with 1E10 gc AAV9-Lucif. or -ATF4. Gapdh serves as a loading control. **k**, **l** Representative micrographs of fluorescent LC3 puncta (**k**) and quantification thereof (**l**) in cultured primary neonatal rat ventricular myocytes 48 h after infection with adenoviruses to overexpress tandem mRFP-GFP-LC3 (Ad-tf-LC3) and ATF4 or βgal expressing control. Yellow dots represent autophagosomes, whereas red dots indicate autolysosomes. Scale bars are 50 μm. *P < 0.0001 vs Adβgal. Number of biologically independent animals or cells analyzed is indicated in each panel. All statistical analysis were performed using two-tailed Student’s *t* test. Error bars are ±standard error of the mean. Source data are provided as a Source Data File.

Like PERK overexpression in the mouse heart, ATF4 overexpression using AAV9-mediated gene transfer beginning at p7 also promoted a profound atrophic phenotype (Fig. 9a). AAV9-ATF4 at a high (0.5E11 Genome Copies (GC)) and low titer (1E10) induced robust protein levels of ATF4 in the heart (Fig. 9b). The highest viral titer led to high mortality by 4 weeks of age, which was not observed in the AAV9-Luciferase injected control littermates. These high levels of ATF4 resulted in reduced heart size, as well as reduced cardiomyocyte surface area of infected, ATF4 positive cardiomyocytes (Fig. 9c–e). Lowering the dose of AAV9-ATF4 (1E10) circumvented mortality yet was still sufficient to induce high levels of ATF4 in the heart as well as a minor but significant elevation of PERK protein levels, suggesting a positive feedback mechanism (Fig. 9b and Supplementary Fig. 7g, h). Low dose of AAV9-ATF4 resulted in atrophy of the heart as shown by a reduced heart size, HW/BW ratio, as well as reduced cardiomyocyte surface area of the ATF4 positive cardiomyocytes (Fig. 9f–i). Western blot analysis confirmed activation of autophagy markers LC3b-II and p62 in the heart as compared to control injected hearts (Fig. 9j and Supplementary Fig. 7i, j). In agreement with our findings in the AAV9-PERK injected hearts, ATF4 overexpression did not alter the levels of ubiquitinated proteins, impact the protein levels of general ER stress markers Armet, BiP, and calreticulin, nor did it alter the protein levels of Thbs1 compared to AAV9-Luciferase injected controls (Fig. 9j and Supplementary Fig. 7k, l). More importantly, adenoviral-mediated overexpression of ATF4 in NRVMs was sufficient to increase autophagosome and autolysosome formation, thus confirming that induction of ATF4 is sufficient to enhance autophagic flux in cardiomyocytes (Fig. 9k, l and Supplementary Fig. 7m). Hence, our data reveal that overexpression of ATF4 drives the induction of autophagy, cardiomyocyte size reduction, and cardiac atrophy as observed with PERK or Thbs1.

## Discussion

Positive and negative effects have been ascribed to the different Thbs proteins in the adult mammalian heart. *Thbs1*^29–31^, *Thbs2*^32–34^, and *Thbs4*^13,35^ gene-deleted mice are viable and overtly normal but are predisposed to greater cardiac injury with disease-associated stimulation, while *Thbs3*^*−/−*^ mice are uniquely protected from injury^9^. Consistent with these findings, we observed greater cardiac hypertrophy with pressure overload stimulation in *Thbs1* ^*−/−*^ mice. In addition, these same mice displayed less fasting-induced cardiac atrophy, together suggesting a role for Thbs1 in regulating adult cardiomyocyte size and ventricular plasticity^1,2^.

Interestingly, while all 5 Thbs matricellular proteins traverse the intracellular secretory pathway and are ultimately secreted, they are also taken back up into cells by the low-density lipoprotein receptor-related protein 1 (LRP1) receptor^36^ and as such, only small amounts of these proteins appear to reside within the extracellular matrix (ECM) of tissues. Such observations suggest functionality of the Thbs proteins from within the secretory pathway, such as the known ability of Thbs1, Thbs3, and Thbs4 to mediate an ATF6α-dependent ER-stress response that expands the vesicular network, alters the levels of membrane attachment complex proteins, and augments the levels of select ECM proteins^8–10,13,21^. Moreover, we have also shown that Thbs4 itself functions as an ER resident chaperone where it can directly refold proteins^9^. Finally, overexpression of a mutant form of Thbs4 that cannot be secreted is pathologic to the heart because it now reduces the content of dystrophin-glycoprotein attachment complexes within the sarcolemma of cardiomyocytes^8^. However, addition of recombinant Thbs3 or Thbs4 to the outside of cells did not affect the transport or total levels of integrins to the sarcolemma of cardiomyocytes, indicating that Thbs3 and Thbs4 work from within the secretory pathway as they are being secreted to alter membrane attachment complexes^9^.

In pancreatic β-cells Thbs1 was shown to activate PERK within the ER leading to NRF2 activation, which then reduced oxidative stress damage to these cells^37^. Here, we determined that the N-terminal LamG domain and C-terminal domain in Thbs1 interact with PERK, which could mediate PERK activation, followed by downstream phosphorylation of eIF2α and induction of ATF4. However, while we observed that Thbs1 activates PERK, our results differ from the work in β-cells in that we failed to find a protective effect^37^. Instead, our studies revealed that Thbs1-mediated activation of the PERK-eIF2α-ATF4 ER stress axis in cardiomyocytes selectively induced autophagy, autophagic flux, and lysosomal protein degradation, and ultimately a lethal atrophic cardiomyopathy.

Increased PERK activity and induction of ATF4 was previously shown to augment the transcription of genes involved in the formation, elongation, and function of the autophagosome, including *becn1*, *Map1lc3b*, multiple autophagy-related genes (*Atg’s*), and *Sqstm1* (p62)^6,7^. Furthermore, hyperactivation of the autophagy-lysosomal system has recently emerged as a central pathway that facilitates cardiac atrophy^28,38–41^, whereas inhibition of autophagy can reverse cardiac atrophy and dysfunction in mouse models of cardiac atrophy^38,39^. In line with these findings, we show for the first time that overexpression of PERK or ATF4 in myocytes of the heart was sufficient to induce autophagy, cardiomyocyte size reduction, and cardiac atrophy, like Thbs1 overexpression. These findings are in line with recent studies in skeletal muscle where activation the PERK-eIF2α-ATF4 pathway was sufficient to promote muscle fiber atrophy^24,42^, whereas genetic deletion of *Atf4* could delay it^42,43^. Finally, the enhanced autophagy and lethal cardiac atrophy with Thbs1 overexpression was largely corrected by deletion of the *Eif2ak3* gene (PERK protein), suggesting a direct causal relationship.

Our findings indicate that Thbs1 functions in a unique manner in cardiac muscle, compared with Thbs2, 3, and 4. Indeed, overexpression of Thbs1, but not Thbs2, or 3 in cultured cardiac myocytes was sufficient to enhance the formation of autophagosomes and increase autophagosome-lysosomes fusion, and thus autophagic flux. Furthermore, in vivo cardiac-specific overexpression of Thbs3 did not lead to increased autophagy or atrophy^9^, and overexpression of Thbs4 in the heart was even protective in response to stress stimulation^13^. Finally, we demonstrated that overexpression of Thbs2 in the heart, which is similar to Thbs1^11^, did not produce a pathologic phenotype or cardiac atrophy.

PERK appears to be a central effector of Thbs1 in the heart given the strong corrective phenotype observed with deletion of *Eif2ak3* in Thbs1 DTG hearts. We also observed that the levels of ubiquitinated proteins and several key components of the ERAD machinery were enhanced with cardiac overexpression of Thbs1. However, this is probably independent of the atrophic mechanism given that some of these same changes were observed in Thbs3 DTG hearts, and given the fact that all Thbs proteins increase ATF6α activity, which is a known regulator of proteostasis in the heart, primarily by inducing genes encoding ER-resident chaperones and ERAD components^13,44–46^. Indeed, we observed comparable changes in ERAD in the Thbs3 and Thbs4 transgenic hearts, but these mice do not develop lethal cardiac atrophy^9,13^. In fact, we show that loss of *Atf6* appeared to exaggerate the atrophic cardiomyopathy in Thbs1 DTG mice, further suggesting that ATF6α is part of an adaptive and protective response. In addition, protein ubiquitination that is a key step in the ERAD pathway^47^ was not altered during cardiac atrophy due to AAV9-mediated PERK or ATF4 overexpression.

Thbs1 is reported to function as an activator of TGFβ^48^, which has been implicated in skeletal muscle cachexia and atrophy^49^. However, generation of a cardiomyocyte-specific Thbs1 overexpressing transgenic mouse which lacked the type-1 repeats that binds TGFβ acted similar to WT Thbs1, each causing lethal atrophic cardiomyopathy. This result suggests that Thbs1 has its pathologic effect in the heart independent of TGFβ. Indeed, we previously generated cardiac-specific transgenic mice expressing an activated and non-latent form of TGFβ that did not impact heart growth or atrophy, but instead caused fibrosis^50^. This suggests that TGFβ is unique in regulating skeletal muscle atrophy directly, but this relationship does not appear to exist in the heart.

The pathologic effect of Thbs1 is also likely independent of angiogenesis in the heart because capillary density was not altered in Thbs1 DTG hearts compared with controls. Indeed, the mutant form of Thbs1 lacking the type-1 repeat region, which harbors the angiogenesis and TGFβ affecting domains, similarly led to lethal cardiomyopathy due to persistent atrophy. Finally, the pathologic effect of Thbs1 was also independent of the previously proposed cell surface receptors CD36 and CD47^51^.

The results presented here reveal a previously undescribed and critical role for Thbs1, PERK, and ATF4 in maintaining protein homeostasis in the heart. More specifically, we identified Thbs1-mediated activation of the PERK-eIF2α-ATF4 ER stress pathway and subsequent induction of unrestrained autophagic protein degradation as a novel pathway central to size control of adult cardiomyocytes in the stressed heart.

## Methods

### Genetically modified mice

All experimental procedures with animals were approved by the Institutional Animal Care and Use Committee (IACUC) of Cincinnati Children’s Medical Center, protocols IACUC 2016-0069 and 2018-0047. The number of mice used in this study reflects the minimum number needed to achieve statistical significance based on experience and previous power analysis. Blinding was performed for some experimental procedures with mice, although blinding was not possible in every instance. Both sexes of mice were used in equal ratios. Animals were kept in the following controlled housing conditions: 21–22 °C, 40–60% humidity and 12-h light/12-h dark cycle.

Cardiomyocyte-specific transgenic mice were generated using the bigenic tetracycline murine α-myosin heavy chain (αMHC) promoter expression vector system^16^. In the absence of doxycycline (Dox) expression is induced, but with Dox administration (200 mg Dox/kg chow, irradiated; Envigo Teklad Diets, #TD.00502) expression is suppressed. Dox in the chow was used to repress transgene expression until weaning (Fig. 2f–i) or after weaning (Fig. 2j–m). Full-length mouse *Thbs1* cDNA was obtained from Open Biosystems (#BC042422), amplified by PCR, and cloned in the Sal1 and HindIII sites of the αMHC responder vector, followed by NotI digestion and gel purification to remove the vector backbone. The Thbs1Δt1 DTG mutant lacking the Thbs1 type1 repeats was created using the In-Fusion HD cloning system (Takara Bio, #639649) and the Thbs1-αMHC responder vector. Thbs2 transgenic mice were generated in the same bi-genic inducible αMHC promoter system and the *Thbs2* cDNA was obtained from Open Biosystems (#BC146676.1), amplified by PCR and cloned in the Sal1 site of the αMHC responder vector, followed by NotI digestion and gel purification to remove the vector backbone. Primer sequences used for cloning are listed in Supplementary Table 1. The final constructs were confirmed by DNA sequencing and injected into newly fertilized oocytes to generate transgenic animals at the University of Cincinnati Transgenic Mouse Core (*C57Bl/6J* background). Mice harboring the αMHC-*Thbs* transgenes were crossed to mice expressing the tetracycline trans-activator (tTA) under the control of the αMHC promoter to generate double transgenic animals (DTG), which were maintained in the *C57Bl/6J* background^16^. Thbs3 inducible cardiac-specific transgenic mice were previously generated in our lab^9^. Mice deficient for *Thbs1*, *Cd36*, *Cd47*, and *Eif2ak3*-loxP targeted (*B6.129S2-Thbs1tm1Hyn/J, B6.129S1-Cd36tm1Mfe/J, B6.129S7-Cd47tm1Fpl/J*, and *Eif2ak3tm1.2Drc/J*) were purchased from Jackson Laboratories and maintained on a *C57Bl6* background. Gene-deleted mice lacking ATF6α protein were obtained from the lab of Kazutoshi Mori (Kyoto University, Kyoto, Japan)^52^. Other mice used in this study included the βMHC-Cre mouse line^53^, mice with cardiomyocyte-specific overexpression of activated calcineurin (ΔCnA TG)^14^ and *Csrp3*^*−/−*^ mice^15^.

For fasting studies, 8-week-old male mice were deprived of food for 48 h with free access to drinking water^54^. To measure autophagic flux in vivo, 8-week-old mice were subject to intraperitoneal injections of bafilomycin A1 (Baf. A1, 2.5 mg/kg (InvivoGen, #tlrl-baf1)), or vehicle control (60% dimethyl sulfoxide in saline). Mice were killed 4 h after Baf. A1 or DMSO injection for further analysis^55^. No human subjects or human tissue was used in this study.

### AAV9 virus production and injection into neonatal mice

Full-length mouse *Atf4* cDNA was obtained from Addgene (plasmid #21845, deposited by Dr. David Ron), amplified by PCR using CloneAMP HiFi PCR premix (Takara Bio, #639298) and cloned into the *Cla1* and *XhoI* sites of pAAV-MCS vector (Cell biolabs, #VPK-410) using the NEBuilder HiFi DNA Assembly Master Mix (New England Biolabs, #E2621). The firefly luciferase cDNA was obtained from Addgene (plasmid #120522, deposited by Dr. Petr Svoboda), amplified by PCR and cloned into the *Cla1* and *XhoI* sites of pAAV-MCS vector (Cell biolabs, #VPK-410) using the NEBuilder HiFi DNA Assembly Master Mix (New England Biolabs, #E2621). Primer sequences used for cloning are listed in Supplementary Table 1. The PERK-Myc insert was obtained from PERK1:WT.9E10.pCDNA (Addgene #21814, deposited by Dr. David Ron) and cloned into CAG-TdTomato AAV backbone by the Howard Hughes Medical Institute (HHMI)-Janelia Viral Tools. Briefly, the PERK-Myc insert was excised by KasI and SphI digestion. New ApaI and SfuI sites were added to the 5′ and 3′ ends of the PERK-Myc insert, respectively, and then cloned into an ApaI-SfuI digested CAG-TdTomato AAV backbone (generated by HHMI-Janelia Viral Tools), resulting in pAAV-CAG-PERK-Myc. The final constructs were confirmed by DNA sequencing. AAV9-CMV-Luciferase, AAV9-CMV-ATF4, and AAV9-CAG-PERK-Myc were produced by the HHMI-Janelia Viral Tools- using a polyethylenimine (PEI) triple transfection protocol into 293 cells, grown under serum-free conditions, purified by two rounds of CsCl density gradient centrifugation and exchanged into storage buffer containing 1x PBS, 5% sorbitol and 350 mM NaCl and subsequently stored at −80 °C until commencing the in vivo experiments. Virus titers (GC/ml) were determined by qPCR targeting the AAV ITRs.

The indicated titers of AAV9-Luciferase, AAV9-PERK or AAV9-ATF4 were injected into the mediastinum of 7-day-old *C57Bl/6J* wild-type mice (Jackson Laboratories, #000664)^56^. Mice were killed and hearts were excised at 4 or 8 weeks of age and the apex was snap frozen in liquid nitrogen for storage at −80 °C. The remainder of the heart was fixed overnight in 4% paraformaldehyde, dehydrated in ethanol, and paraffin embedded for further histological and immunohistochemical analysis.

### Adenoviruses, cell culture, and in vitro autophagic flux assay

Adenoviruses harboring Thbs1, Thbs3, tandem mRFP-GFP-LC3 (Ad-tf-LC3; gift from Dr. J. Sadoshima of the University of Medicine and Dentistry of New Jersey, Newark), and β-galactosidase (βgal) expressing control were previously generated and validated^9,13,25^. The *Thbs2* and *Atf4* cDNA were amplified by PCR for insertion into the pShuttle-CMV vector (Agilent Technologies #240007) to generate recombinant adenoviruses according to the manufacturer’s instructions^13^.

Primary neonatal rat ventricular myocytes (NRVMs) were prepared from 1- to 2-day-old Sprague-Dawley rat pups^9^. Briefly, neonatal hearts were collected, the atria were removed and the ventricles were minced in Hanks’ Balanced Salt Solution (HBSS) prior to five rounds of enzymatic digestion using 0.05% pancreatin (Sigma) and 84 units/ml of collagenase (Worthington, Lakewood, NJ). Next, cells were collected by centrifugation (500×*g* for 5 min at 4 °C) and resuspended in HyClone Medium 199/ Earle’s Balanced Salt Solution (EBSS) (ThermoScientific, SH30253FS) supplemented with 2% fetal bovine serum (Sigma-Aldrich, F2442) and 1x penicillin-streptomycin (Cellgro 30-0002-CI, Mediatech, Corning Life Sciences, Tewksbury, MA). The cells were then differentially plated for 1 h on uncoated culture dishes to reduce fibroblasts. Finally, NRVMs were plated on gelatin-coated cell culture dishes and maintained in the culture medium described above.

The in vitro autophagic flux assay was performed as described with modifications^25^. Briefly, NRVMs were plated with a density of 50 × 10^3^ cells per well in Ibidi μ-slide eight-well dishes (Ibidi USA, Inc. Madison, WI, Cat# 80826) and cultured in HyClone Medium 199/EBSS (ThermoScientific, SH30253FS) supplemented with 2% fetal bovine serum (Sigma-Aldrich, F2442) and 1x penicillin-streptomycin (Cellgro 30-0002-CI, Mediatech, Corning Life Sciences, Tewksbury, MA). The next day, NRVMs were infected with recombinant adenoviruses to overexpress tandem mRFP-GFP-LC3 (Ad-tf-LC3) with either Thbs1, Thbs2, Thbs3, ATF4, or βgal control for 3 h in serum-free media after which they were switched back to culture media supplemented with 2% fetal bovine serum. Forty-eight hours later cells were washed with 1x PBS, fixed with 4% paraformaldehyde, washed again, mounted with Ibidi mounting medium (Ibidi USA, Inc. Madison, WI, Cat# 50001), and visualized using a Nikon A1 confocal laser microscope system equipped with 40x H_2_O objective (NA = 1.15). All imaging was performed under identical conditions using NIS Elements Advanced Research (AR) microscope imaging software (Nikon Instruments Inc. Melville, NY). The tf-LC3 is a modified LC3 (microtubule-associated protein 1 light chain 3) with N-terminal fusion of an GFP and a mRFP in tandem^25^. While imaging, tf-LC3–containing autophagosomes are visualized as yellow puncta because both the GFP and mRFP fluoresce. However, the tf-LC3-labeled autolysosomes emit only red fluorescence from mRFP because the acidic environment in autolysosomes quenches GFP^25^. The number of mRFP-GFP double fluorescent LC3 puncta (autophagosomes) and mRFP fluorescent LC3 puncta (autolysosomes) per NRVM was determined using NIS Elements Advanced Research (AR) microscope analysis software (Nikon Instruments Inc. Melville, NY). In parallel, protein lysates were prepared from NRVMs that were cultured in 100 mm cell culture dishes (Thomas Scientific, Swedesboro, NJ, Cat # 1156F06) to validate adenoviral-mediated overexpression of Thbs1, Thbs2, Thbs3, and ATF4 as described in the section ‘Protein isolation and western blotting’. All experiments described above were performed in triplicate.

### Protein isolation and western blotting

Hearts were excised, immediately frozen in liquid nitrogen and stored at −80 °C until further use. Cultured primary NRVMs were washed three times with ice-cold 1x PBS, collected, centrifuged at 500×*g* at 4 °C and the pellet was snap frozen in liquid nitrogen and stored at −80 °C until further use. General protein samples were prepared in an ice-cold modified radio-immunoprecipitation assay (RIPA) buffer (1% Triton X-100, 1% sodium deoxycholate, 0.1% sodium dodecyl sulfate (SDS), 50 mM Tris-HCl, pH 7.4, 150 mM NaCl) supplemented with Halt^TM^ Protease and Phosphatase single-use inhibitor cocktail (ThermoFisher Scientific, #78442). Samples were sonicated (SP Scientific, Warminster, PA, VirSonic 60, power setting 3 for three times 10 s), lysates were cleared by centrifugation at 20,500×*g* for 14 min at 4 °C and stored at −80 °C.

ECM proteins were extracted from frozen whole heart ventricles using a subcellular protein fractionation kit for tissues (ThermoFisher Scientific, Cat. #87790) according to the manufacturer’s instructions. In brief, heart tissue was washed in ice-cold 1x PBS, minced, and homogenized in ice-cold cytoplasmic extraction buffer (CEB). At this point, a 20 μl aliquots of ‘total protein extracts’ (TOTAL; Fig. 3g) were collected and kept separately. Next, the samples were alternatingly centrifuged and incubated in a series of buffers to sequentially remove cytoplasmic proteins (buffer ‘CEB’), plasma, mitochondria, and ER/Golgi membrane proteins (buffer ‘MEB’), soluble and chromatin-bound nuclear proteins (buffer ‘NEB’ and ‘NEB + CaCl_2_ and Micrococcal Nuclease’), cytoskeletal proteins (buffer ‘PEB’). The remaining ECM-enriched pellets were considered ‘ECM extracts’ (Fig. 3g), and were resuspended in 6 M urea, 0.1% SDS, and 1% dithiothreitol (DTT).

In general, protein concentrations were determined using a Direct Detect infrared spectrometer (Millipore Sigma). Protein concentrations of ECM fractions were determined using NanoDrop A280 measurements (Denovix, Wilmington, DE, Cat. # DS-11 FX + ). All protein preparations were mixed with 5x Laemmli loading buffer and heated at 95 °C for 10 min prior to loading on SDS-polyacrylamide gel electrophoresis (PAGE) or Phos-tag (*Phos*-*tag*^™^ acrylamide) SDS-PAGE gels (see below). For standard Western blots, a wet transfer method to polyvinylidene fluoride (PVDF) membranes with a 0.45-μm pore size (EMD Millipore, #IPFL00010) or PVDF membranes with a 0.2-μm pore size (EMD Millipore, #ISEQ20200) in case of LC3b blotting was utilized.

A Phos-tag based system was used to investigate the activation status of PERK due to the lack of reliable commercially available phospho-specific antibodies^57^. Phos-tag SDS-PAGE gels were run as previously described with modifications^57^. Briefly, Phos-Tag™ acrylamide compound (FUJIFILM Wako Chemicals, Cat. # 304-93521) was incorporated in 6% Bis acrylamide gels according to the manufacturer’s instructions. After electrophoresis at 115 V, Phos-tag gels were incubated for 10 min in transfer buffer containing 1 mM ethylenediaminetetraacetic acid (EDTA) followed by 10 min in transfer buffer without methanol or EDTA. Next, proteins were transferred to nitrocellulose membranes with a 0.2 μm pore size (Bio-Rad, Cat. # 162-0112) at 85 V for 1.5 h using the standard wet transfer method. Precision Plus Protein™ Dual Color Standards was utilized as protein marker for all regular western blots (Bio-Rad, Car. #1610374), whereas Wide-View™ Pre-stained Protein Size Marker III was used for Phos-tag gels (FUJIFILM Wako Chemicals, Cat. #230-0246).

Immunoblots were performed using appropriate primary antibodies and fluorescent conjugated secondary antibodies (LI-COR, IRdye 800CW Goat anti-Mouse #926-32350, IRdye 800CW Goat anti-Rat #926-32219, IRdye 800CW Goat anti-Rabbit #926-32211, IRdye 680RD Goat anti-Mouse #926-68072, IRdye 680RD Goat anti-Rabbit #925-68073, IRdye 800CW Donkey anti-Goat #926-32214, IRdye 680RD Donkey anti-Goat #926-68074, IRdye 680RD Donkey anti-Mouse #926-68072; all at 1:3000) in combination with an Odyssey CLx Infrared Imaging System (LI-COR). Western blot band intensities were quantified using the Li-Cor Image Studio software.

Primary antibodies used were: Armet (Abcam, #ab67271 at 1:1000), p-AKT ser473 (Cell Signaling Technology, #9271 at 1:1000), AKT (Cell Signaling Technology, #9272 at 1:1000), ATF4 (Cell Signaling Technology, #11815; at 1:1000), ATF6α (SAB SignalWayAntibody LLC, #SAB24383; at 1:1000), BiP (Sigma, #G9043 at 1:1000), CD36 (Abcam, #ab133625 at 1:1000), CD47 (Abcam, #ab108415; at 1:1000), calreticulin (Cell Signaling Technology, #2891; at 1:1000), Derlin-1 (Abcam, #ab176732; at 1:1000), Derlin-3 (Sigma, #D2194; at 1:1000), DNAJC3 (Abcam, #ab227140 at 1:1000), Edem1 (LifeSpan Biosciences, #LS-C80983), p-eIF2α ser51 (Cell Signaling Technology, #9721; at 1:1000), eIF2α (Cell Signaling Technology, #9722; at 1:1000), Herpud1 (Cell Signaling Technology, #26730; at 1:1000), IRE1α (Cell Signaling Technology, #3294; at 1:1000), p-IRE1α ser724 (Abcam, #ab48187; at 1:1000), LC3b (Cell Signaling Technology, #3868 at 1:1000), p-mTOR ser2448 (Cell Signaling Technology, #2971 at 1:1000), mTOR (Cell Signaling Technology, #2972 at 1:1000), p62 (Sigma, #p0067 at 1:1000), PERK (Cell Signaling Technology, #3192; at 1:1000), p-P70S6K thr389 (Cell Signaling Technology, #9205; at 1:1000), P70S6K (Cell Signaling Technology, #9202; at 1:1000), SEL1L (Sigma, #S3699; at 1:1000), Thbs1 (R&D Systems, #AF3074; at 1:500), Thbs2 (BD Bioscience, #611150; at 1:1000 in Fig. 2k and R&D Systems, #MAB1635; at 1:500 in SupFig. 3g), Thbs3 (Proteintech, #19727-1-AP; at 1:100), Ubiquitin (Santa Cruz Biotechnology, #sc-8017; at 1:200), VCP/P97 (Abcam, #ab36047; at 1:1000), and Vinculin (Sigma, #V9131; at 1:1000) or glyceraldehyde 3-phosphate dehydrogenase (Gapdh; Fitzgerald, #10R-G109A at 1:10000) served as loading controls. Coomassie Brilliant Blue staining of the electrophoresed gels was performed according to the Abcam website (https://www.abcam.com/protocols/transfer-and-staining-of-proteins-in-western-blot) to visualize protein loading of the ECM fractions. All antibodies were authenticated for proper specificity, in some cases using tissues or cells from gene-deleted mice or using authentication data provided by the vendor for each antibody. Uncropped images of all western blots presented in this study can be found in the Source Data File.

### Immunoprecipitation and GST Pull-Down

Protein extracts from tTA cont. and Thbs1 DTG hearts were generated by homogenizing in immunoprecipitation (IP) lysis buffer (50 mM Tris-HCl pH 7.4, 150 mM NaCl, 1% Triton X-100) supplemented with Halt^TM^ Protease and Phosphatase single-use inhibitor cocktail (ThermoFisher Scientific, #78442), followed by rotation at 4 °C for 2 h and clarification by centrifuging samples at 15,000×*g* for 20 min at 4 °C. Protein concentration was determined by Bradford. PERK IPs were performed with 1 mg of lysate incubated with 2 µg of primary antibody (Proteintech, #20582-1AP) or the corresponding IgG (Cell Signaling Technology, #2729) overnight at 4 °C with rotation. Next, magnetic beads (Dynabeads^TM^ Protein G, Invitrogen, #1003D) were washed three times in lysis buffer then added to samples per manufacturer’s recommendation (8 μg IgG/mg beads) and rotated for an additional hour at 4 °C. Beads were then washed three times in wash buffer (50 mM Tris-HCl pH 7.4, 150 mM NaCl, 0.1% Triton X-100) supplemented with Halt^TM^ Protease and Phosphatase single-use inhibitor cocktail. Finally, beads were resuspended in 2X sample buffer and heated at 95 °C for 10 min. Thbs1 IPs were performed simultaneously using a Biotin-labeled monoclonal Thbs1 primary antibody (Invitrogen, #MA5-13395), or mouse IgG (Santa Cruz Biotechnology, #sc-2025), and 10 μg/mg Dynabeads^TM^ M-280 Streptavidin (Invitrogen, #11205D). Immunoblots were performed as described in the section ‘Protein isolation and western blotting’ using primary antibodies against PERK (Proteintech, 20582-1AP; at 1:1000), Thbs1 (R&DSystems, #AF3074; at 1:500), or Vinculin (Sigma, #V9131; at 1:1000) as a loading control.

GST-Thbs1 fusion plasmids were provided by Dr. Jack Lawler, Beth Israel Deaconess Medical Center^58^. GST empty vector and GST-Thbs1 fusion plasmids corresponding to the Thbs1 N-terminus (lamG domain), aa 1–240; procollagen (PC) domain, aa. 278–355; type 1 repeat (T1) domain, aa. 385–522; type 2 repeat (T2) domain, aa. 559 to 669; type 3 repeat (T3) domain, aa. 784–932; and the Thbs1 carboxy-terminal domain (CTD), aa. 926–1152 (see diagram Fig. 4d) were transformed into Bl21 Star (DE3) competent *E. coli*. Next, cultures were grown for protein expression in LB-Amp at 37 °C to an OD_600_ of 0.6 at which time 1 mM isopropyl β-d-1-thiogalactopyranoside (IPTG) was added, and cultures were grown for 4 more hours. Cultures were then centrifuged at 3000×g for 20 min; the pellet was washed by resuspending it in 10 ml of ice-cold 1x PBS and recentrifuged again. Pellets were snap frozen in liquid N_2_ and stored at −80 until further use. Frozen pellets were thawed, resuspended in suspension buffer (10 mM Tris-HCl pH 8.0, 1 mM EDTA, 150 mM NaCl), and lysed by addition of 1 mg/ml lysozyme and subsequent incubation for 15 min at 4 °C with gentle agitation. DTT (10 mM) and Sarkosyl (1.22%) were added followed by sonication for a total of one minute. Lysates were then centrifuged at 21,000×*g* for 20 min and the pellet was discarded. Triton-X 100 was added to the supernatant for a final concentration of 2% and incubated for 30 min on ice. Glutathione sepharose (Glutathione Sepharose 4B, GE Healthcare, #17-0756-01) was washed three times in suspension buffer with Sarkosyl (0.7%), DTT, and Triton X-100. Washed GST-sepharose (500 µl packed) was added to each sample and incubated for a minimum of 2 h at 4 °C with agitation. Sepharose was pelleted by centrifugation at 500×*g* for 5 min at 4 °C and washed three times in resuspension buffer with detergent followed by a final wash in 1x PBS. The protein bound glutathione sepharose samples were then resuspended in crude lysates from primary neonatal rat ventricular myocytes (NRVMs; 2 ug/sample) and rotated at 4 °C overnight. NRVM lysates were prepared as described for IPs. The samples were then washed and prepared for immunoblotting as described above. Immunoblots were performed as described in the section *‘Protein isolation and Western blotting’* using primary antibodies against PERK (Cell Signaling Technology, #3192; at 1:1000). Purity of isolated GST empty vector and GST-Thbs1 fusion constructs were confirmed by Coomassie Brilliant Blue staining of SDS-PAGE gels.

### TGFβ ELISA and proteasome activity assay

Enzyme-linked immunosorbent assays (ELISA) were performed on heart homogenates using a kit for TGF-β1 purchased from R&D Systems (SMB100B) and according to the manufacturer’s instructions^59^. The chymotrypsin-like activity of the 20S proteasome in ventricular extracts was determined using a commercially available proteasome activity kit following the manufacturer’s instructions (Abcam, Cat. #ab107921).

### Pressure overload and echocardiography

Eight- to ten-week-old mice of the relevant genotypes were subjected to transverse aortic constriction (TAC) or sham surgical procedure^60^. Briefly, the transverse aortic arch was visualized by performing a median sternotomy. A 7-0 silk ligature was then tied around the aorta and a 27-gauge wire to control the degree of constriction between the right branchiocephalic and left common carotid artery. Next, the wire was removed to generate a defined reduction in lumen area. Doppler echocardiography was performed on mice after TAC to ensure equal pressure gradients across the aortic constriction. Mice with pressure gradients of <45 mmHg were excluded from the results, as this indicated an unsuccessful surgery. Both sexes of mice were used for TAC surgeries. Mice were given buprenex (Henry Schein, #055175) as pain relief at a final concentration of 0.05 mg/ml by subcutaneous injection. Mice were then transferred to 30 °C incubators and monitored while in recovery, and any mouse displaying signs of distress was removed from the study in accordance institutional guidelines approved by the IACUC of Cincinnati Children’s Hospital. Echocardiography was performed and analyzed in a blinded manner on mice after isoflurane inhalation (2% to effect) for anesthesia using a Sonos 5500 ultrasound instrument (Hewlett Packard) with a 15-MHz microprobe and measurements were taken in M-mode in triplicate for each mouse and averaged.

### Electron microscopy

Hearts of anesthetized mice were perfused with relaxing buffer (0.15% sucrose, 5% dextrose, 10 mM KCl in 1x PBS) for 3 min and then perfused with fixation buffer (1% paraformaldehyde, 2% glutaraldehyde in 100 mM sodium cacodylate pH 7.4), then fixed overnight in fixation buffer and post-fixed in 1% OsO_4_ for 2 h. Ultrathin sections of all tissues were counterstained with uranyl acetate and lead salts. Images were obtained using a 7600 transmission electron microscope (Hitachi) connected to a digital camera (AMT, Biosprint16).

### Histological analysis, and immunohistochemistry

Heart tissue was fixed overnight in 10% formaldehyde, dehydrated in ethanol and paraffin embedded. Tissue was sectioned at 5 μm and stained with either hematoxylin and eosin (H&E) or Masson’s trichrome. Images were captured with a stereo microscope (Leica Microsystems, #M165FC) equipped with a digital camera (Leica Microsystems, #DFC310 FX) and the Leica Application Suite. General immunohistochemistry and co-labeling of Thbs1 and BiP was performed on paraffin embedded tissue sections, which were rehydrated and heated in 1x antigen retrieval CITRA (BioGenex, # HK086-9K). For co-labeling of tissue sections with Thbs1 and Vimentin, freshly harvested hearts were embedded in optimal cutting temperature (O.C.T) compound (VWR, #25608-930), frozen in liquid nitrogen and tissue sections were cut at a thickness of 10 μm. Afterwards, both paraffin- and cryo-tissue sections were rinsed in 1x PBS, permeabilized for 2 min in 0.1% triton X-100/PBS and incubated for 1 hour at room temperature in blocking buffer (1x PBS, 5% goat serum, 2% BSA). Primary antibody incubations were performed overnight at 4 °C for Thbs1 (ThermoFisher Scientific, #MA5-13395 at 1:200 for Fig.1b, c; R&D Systems, #AF3074; at 1:200 for Fig.1f), ATF4 (Cell Signaling Technology, #11815 at 1:200), vimentin (Abcam, #ab45939 at 1:200), BiP (Sigma, #G8918 at 1:200), LC3b (Cell Signaling Technology, #2775 at 1:100), and PERK (Santa Cruz Biotechnology, #sc-9477; at 1:50). Appropriate Alexa Fluor-488 (ThermoFisher Scientific, Goat anti-Mouse IgG, #A-11029; Donkey anti-Goat IgG, #A-11055; Goat anti-Rabbit IgG, #A11008), and Alexa Fluor-568 (ThermoFisher Scientific, Goat anti-Mouse IgG, #A-11031; Donkey anti-Rabbit IgG, #A-10042; Donkey anti-goat IgG, #A-11057) secondary antibodies at 1:400 in blocking buffer were applied for 1 h at room temperature and subsequently for 10 min with DAPI (4′,6-diamidino-2-phenylindole) nuclear DNA stain at 1:10.000 (ThermoFisher Scientific, #D1306). Staining with wheat germ agglutinin (WGA) conjugated to fluorescein isothiocyanate (FITC; Lectin from Triticum vulgaris, Millipore Sigma, #L4895 at 1:100 or WGA Alexa Fluor 647 conjugate (Molecular Probes, #w32466 at 1:100) was done for 1 h at room temperature to visualize the membranes. For all immunostains included in this study, sections were incubated with the appropriate secondary antibodies alone as a control for specificity.

All sections were mounted in Vectashield Hard Set (Vector Laboratories, #H-1400) to prevent photo bleaching and visualized using a Nikon A1 + confocal laser microscope system (Nikon Instruments) equipped with 40x H_2_O objective (Nikon Instruments, NA = 1.15). All imaging and analyses were performed under identical conditions using NIS Elements Advanced Research microscope imaging software (Nikon Instruments).

Quantification of cross sectional area (CSA) was performed on at least 4 random pictures of the left ventricle free wall. A minimum of 200 cardiomyocytes were analyzed per animal and per experiment. More specifically, quantitative analysis of AAV9-Lucif. versus AAV9-PERK positive (Pos.) and negative (Neg.) CSA was determined by PERK and WGA-FITC staining on cardiac sections at 8 weeks of age (see above); whereas quantitative analysis of CSA of AAV9-Lucif. versus AAV9-ATF4 positive cardiomyocytes was determined by ATF4 and WGA-FITC staining of cardiac histological sections at 4 weeks of age (see above).

Capillary counts were analyzed on cryosections, co-stained with biotinylated isolectin B4 (Vector Laboratories, #B-1205) and WGA-FITC (Lectin from Triticum vulgaris, Millipore Sigma, #L4895 at 1:100) for 1 h at room temperature, followed by treatment with streptavidin, Alexa 594 conjugate (ThermoFisher Scientific, #S11227 at 1:1000) for 1 h at room temperature to visualize isolectin B4. Pictures were taken with a Nikon A1 confocal microscope. Three random pictures of the left ventricle free wall were taken from 3 sections at different levels for quantitative analysis.

TUNEL staining was performed with an in situ TUNEL detection kit (Roche, #12156792910), and according to the manufacturer’s instructions^28^. Cryosections were then co-stained with isolectin B4 (Vector Laboratories, #B-1205) followed by treatment with streptavidin, Alexa 594 conjugate (ThermoFisher Scientific, #S11227 at 1:1000) for 1 h at room temperature to visualize isolectin B4. Pictures were taken with a Nikon A1 confocal microscope. Three random pictures of the left ventricle free wall were taken from three sections at different levels for quantitative analysis. For endothelial cell apoptosis, isolectin B4/TUNEL/DAPI cells were counted.

An EdU assay was used to determine endothelial cell proliferation^61^. Briefly, mice were treated with EdU (Santa Cruz Biotechnology, #61135-33-9) dissolved in 1x PBS by i.p. injection (5 μg/g of body weight). 24 h after EdU injection, hearts were fixed in 4% paraformaldehyde and frozen for immunohistochemical analysis. Cryosections were co-stained with anti-CD31 (BD Biosciences, #553370), then sections were incubated with a 1:500 dilution of goat anti-rat Alexa Fluor 568 secondary antibodies (A-11077, Life Technologies), followed by EdU staining using a Click-iT EdU Alexa Fluor 488 Imaging Kit (C10641, Life Technologies). Pictures were taken with a Nikon A1 confocal microscope. Three random pictures of the left ventricle free wall were taken from three sections at different levels for quantitative analysis. For endothelial cell proliferation, CD31/EdU/DAPI cells were counted.

### RNA isolation and quantitative reverse-transcriptase PCR

RNA was isolated from hearts using RNeasy Fibrous Tissue Kit (QIAGEN, #74704) according to the manufacturer’s instructions. Synthesis of cDNA was performed using Superscript III First Strand Kit (Invitrogen, #18080-051) with random hexamer primers according to the manufacturer’s instructions. Quantitative real-time PCR was performed using SsoAdvanced SYBR Green (Bio-Rad, #6090) on a CFX96 Touch Real-Time PCR System (Bio-Rad). Primer sequences are listed in Supplementary Table 2. *Rpl13* (ribosomal Protein L13) was used as a reference for relative quantification.

### Statistics and reproducibility

Results are presented in all cases as mean ± SEM. Statistical analysis was performed using Graphpad Prism 9.0.0 (Graphpad Software). Individual variable comparison was analyzed using two-tailed Student’s *t* test, *p*-values <0.05 were considered significant. Comparison of multiple groups was performed using one-way analysis of variance (ANOVA) with Tukey multiple comparisons test, p-values <0.05 were considered significant. Comparison of survival curves was performed using a two-tailed log-rank (Mantel-Cox) test, p-values <0.05 were considered significant. The representative data shown as western blot photograph and microscopy images were obtained from at least three independent experiments or at least three mice per group (for Figs. 1a–g; 2a, k; 3g, j; 4a–d(lower panel); 5b, c, e, f; 6a; 7a, f; 8b–d, g; and 9b–d, f, h, j and Supplementary Figs. 1a, d; 2d; 3d–i; 4; and 7f, l–m). Data generated with cultured primary neonatal rat ventricular myocytes (Figs. 5g–i, and 9k, l) was derived from 3 independent experiments.

### Reporting summary

Further information on research design is available in the Nature Research Reporting Summary linked to this article.

## Supplementary information

Supplementary Information

Reporting Summary

## Data Availability

All original data underlying selected data shown in the figures and supplemental figures are available from the corresponding author upon reasonable request. Source data are provided with this paper.

## Source data

Source Data
