# Supplementary material for: Thbs1 induces lethal cardiac atrophy through PERK-ATF4 regulated autophagy
Source: Nat Commun. 2021 Jun 24;12:3928. doi: 10.1038/s41467-021-24215-4 (PMC8225674; doi:10.1038/s41467-021-24215-4)
Supplement: Supplementary file 1 — Supplementary Information [file 41467_2021_24215_MOESM1_ESM.pdf]

Supplementary information

Thbs1 induces lethal cardiac atrophy through PERK-ATF4 regulated autophagy

Davy Vanhoutte, [1§] Tobias G. Schips,[1#§] Alexander Vo,[1] Kelly M. Grimes,[1] Tanya A. Baldwin,[1] Matthew J. Brody,[1,2] Federica Accornero,[1,3] Michelle A. Sargent,[1] Jeffery D. Molkentin[1,4,*]

[1] Department of Pediatrics, University of Cincinnati, Cincinnati Children's Hospital Medical Center, Cincinnati, OH, 45229, USA.
[2] Department of Pharmacology, University of Michigan, Ann Arbor MI 48109, USA
[3] Department Cell Biology and Physiology, The Ohio State University, Columbus OH 43210, USA
[4] Howard Hughes Medical Institute, Cincinnati Children's Hospital Medical Center, Cincinnati, OH, 45229, USA.

[#] Present address: Janssen Pharmaceuticals, Spring House, PA, 19477, USA.
[§]These authors contributed equally to this work

Supplementary Figures 1-7
Supplementary Table 1 and 2

Supplementary Figure 1

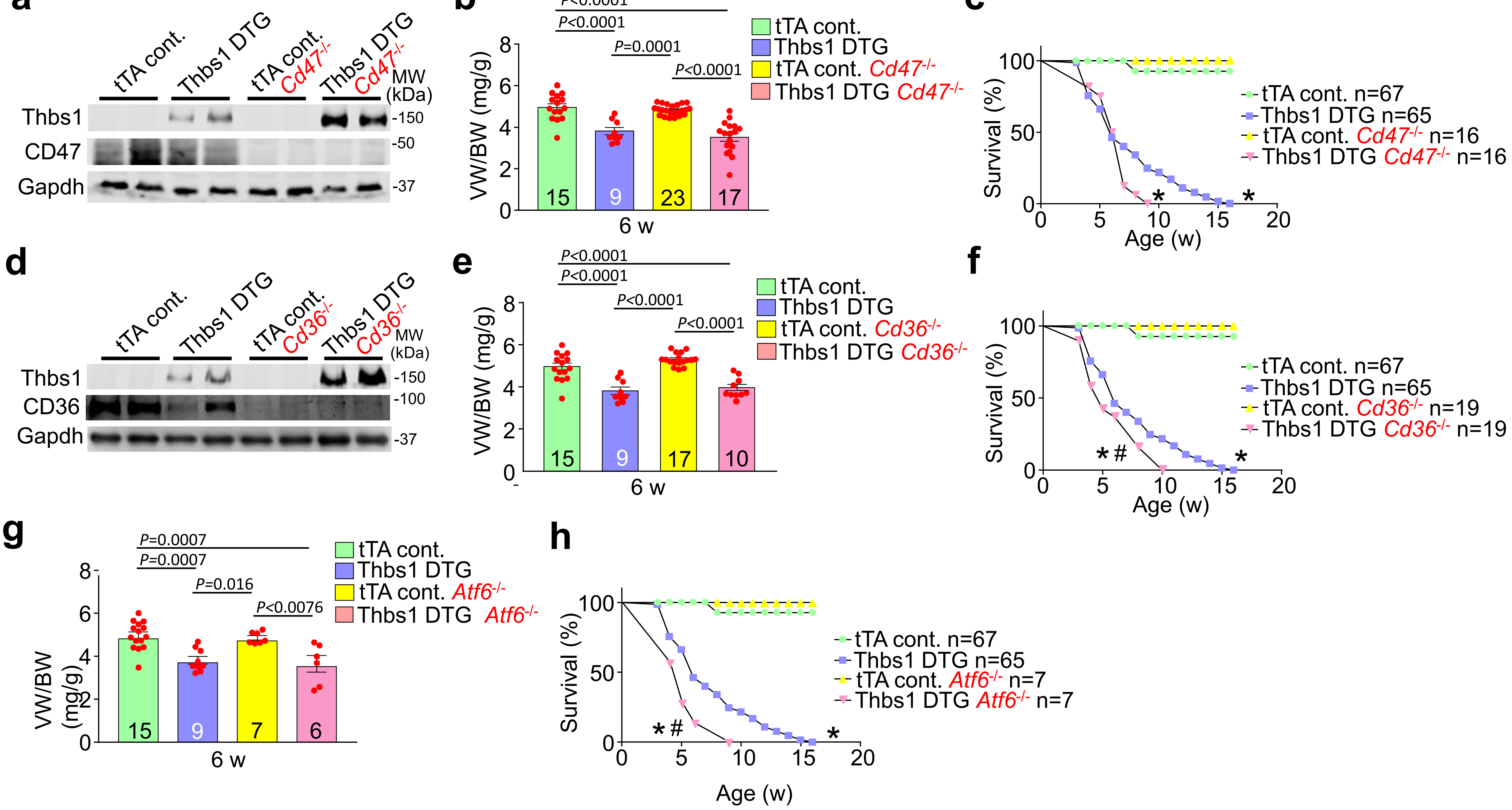

Supplementary Figure 1. **Loss of *Cd47*, *Cd36*, or *Atf6* does not rescue Thbs1-mediated cardiac atrophy.**

a, Western blots for Thbs1, CD47, and Gapdh as a loading control from heart tissue of tTA cont., Thbs1 DTG, tTA cont. *Cd47*$^{-/-}$, and Thbs1 DTG *Cd47*$^{-/-}$ mice at 6 weeks (w) of age. **b**, Ventricular weight-to-body weight (VW/BW) ratio at 6 weeks of age in the indicated groups of mice. **c**, Kaplan-Meier survival plot in the indicated groups of mice; $*P < 0.0001$ vs tTA cont. and *Cd47*$^{-/-}$. **d**, Western blots for Thbs1, CD36, and Gapdh as a loading control from heart tissue of tTA cont., Thbs1 DTG, tTA cont. *Cd36*$^{-/-}$, and Thbs1 DTG *Cd36*$^{-/-}$ mice at 6 weeks of age. **e**, VW/BW ratio at 6 weeks of age in the indicated groups of mice. **f**, Kaplan-Meier survival plot in the indicated groups of mice; $*P < 0.0001$ vs tTA cont. and *Cd36*$^{-/-}$; and $\#P$=0.0339 vs Thbs1 DTG. **g**, VW/BW ratio at 6 weeks of age of tTA cont., Thbs1 DTG, tTA cont. *Atf6*$^{-/-}$, and Thbs1 DTG *Atf6*$^{-/-}$ mice. **h**, Kaplan-Meier survival plot of the indicated groups of mice; $*P < 0.0001$ for Thbs1 DTG vs tTA cont., $*P = 0.0575$ for Thbs1 DTG vs and tTA cont. *Atf6*$^{-/-}$; $*P < 0.0001$ for Thbs1 DTG *Atf6*$^{-/-}$ vs tTA cont., $*P$ =0.0063 for Thbs1 DTG *Atf6*$^{-/-}$ vs tTA cont. *Atf6*$^{-/-}$and $\#P$=0.0168 for Thbs1 DTG *Atf6*$^{-/-}$ vs Thbs1 DTG. Statistical analysis was performed using one-way ANOVA and Tukey multiple comparisons test for panels “b, e, and g”, or two-tailed log-rank test for panels “c, f, and h”. All error bars are +/− standard error of the mean. The number of biologically independent animals analyzed is indicated on the histograms. The survival data shown in panels "c", "f" and "h" are the same for the tTA control and Thbs1 DTG mice shown in Fig 2e and 3i (same strain and ages and sex ratio mix). Source data are provided as a Source Data File.

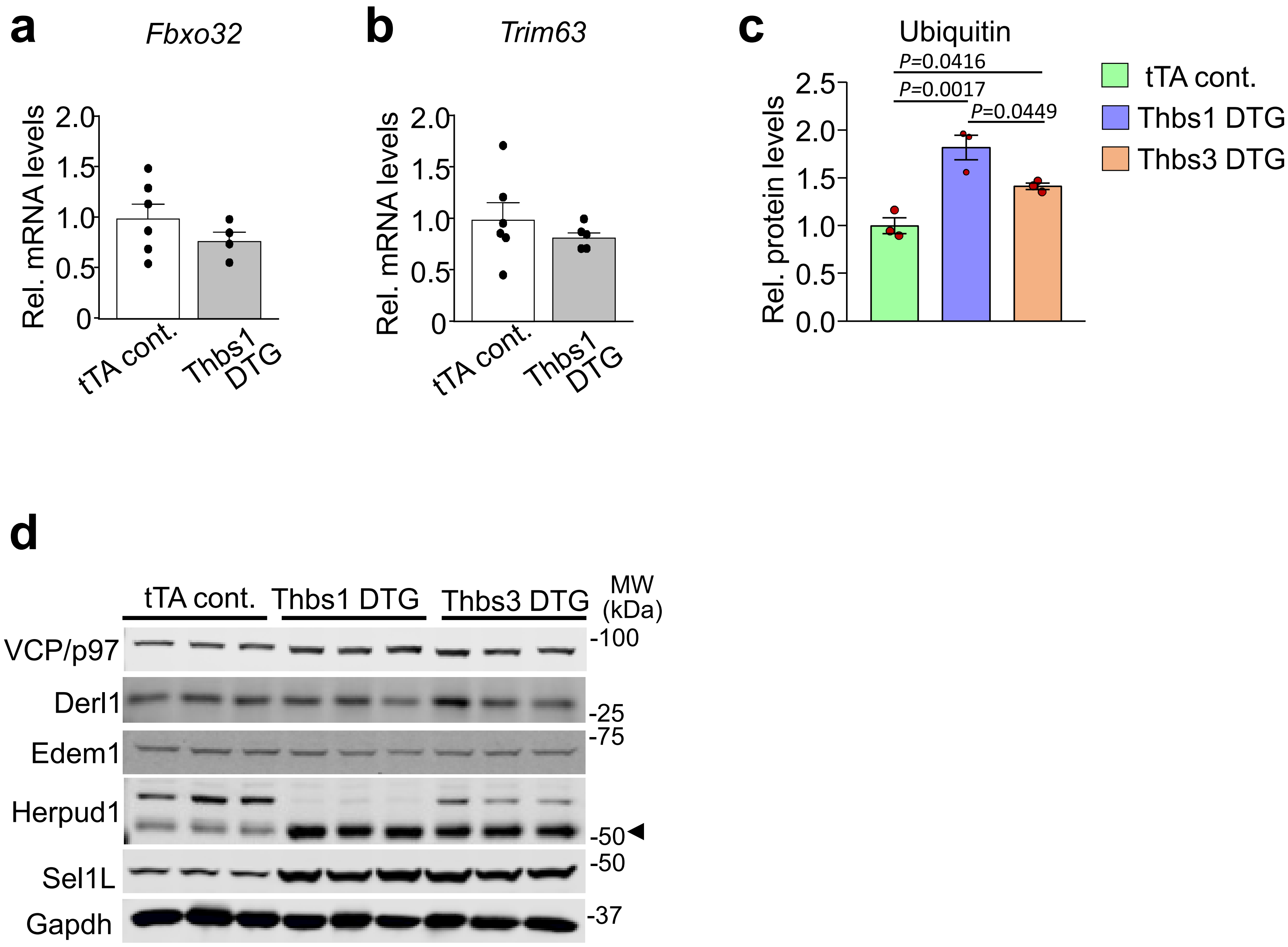
a
Fbxo32
Rel. mRNA levels
2.0
1.5
1.0
0.5
0
tTA cont.
Thbs1 DTG
b
Trim63
Rel. mRNA levels
2.0
1.5
1.0
0.5
0
tTA cont.
Thbs1 DTG
c
Ubiquitin
P=0.0416
P=0.0017
P=0.0449
Rel. protein levels
2.5
2.0
1.5
1.0
0.5
0.0
tTA cont.
Thbs1 DTG
Thbs3 DTG
d
tTA cont.
Thbs1 DTG
Thbs3 DTG
MW (kDa)
VCP/p97
Derl1
Edem1
Herpud1
Sel1L
Gapdh
-100
-25
-75
-50
-50
-37

Supplementary Figure 2. **Evaluating the ubiquitin-proteasome system and ERAD pathway in Thbs1 overexpressing hearts.**

a-b, Quantitative RT-PCR for *Fbxo32* (Atrogin-1) and *Trim63* (MuRF1) mRNA isolated from hearts of tTA cont. (n=6 biologically independent animals) and Thbs1 DTG mice (n=4 biologically independent animals) at 6 weeks of age. Data are represented as fold expression over tTA cont. Error bars are +/− standard error of the mean. **c**, Relative levels of ubiquitin-conjugated proteins determined by Western blot analysis on hearts of tTA cont., Thbs1 DTG and Thbs3 DTG mice and as shown in Fig. 5b (n=3 biologically independent animals per genotype). *P*-values are shown on the graph. Statistical analysis was performed using one-way ANOVA and Tukey multiple comparisons test. Error bars are +/− standard error of the mean. **d**, Representative Western blots for proteins involved in ERAD, including VCP/p97, Derlin-1 (Derl1), Edem1, Herpud1(arrowhead), and Sel1L on protein extracts from heart tissue of tTA cont., Thbs1 DTG and Thbs3 DTG mice at 4 weeks of age. Gapdh serves as a tissue processing and loading control. Source data are provided as a Source Data File.

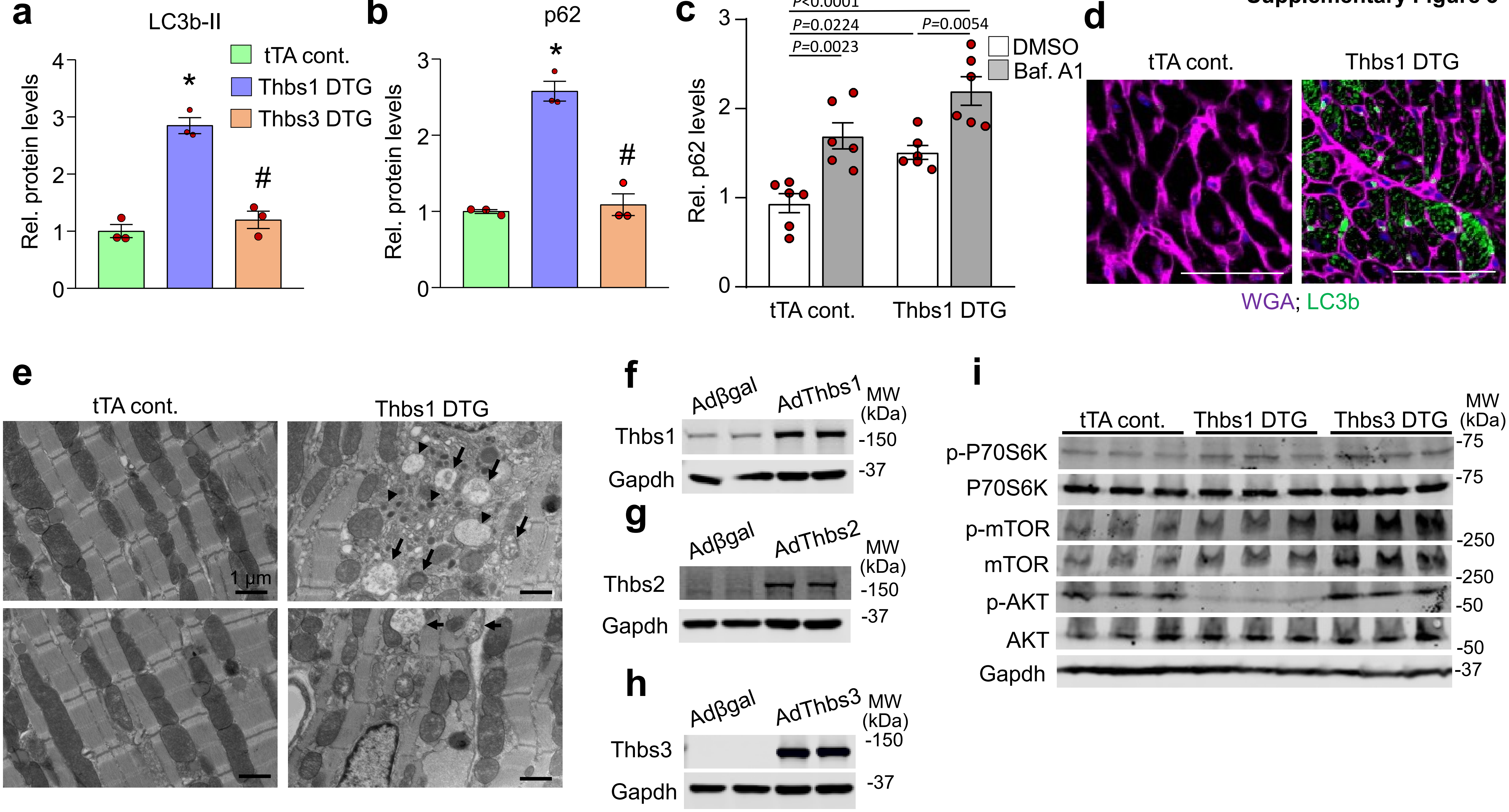
a
LC3b-II
tTA cont.
Thbs1 DTG
Thbs3 DTG
Rel. protein levels
*
#
b
p62
Rel. protein levels
*
#
c
P<0.0001
P=0.0224
P=0.0054
P=0.0023
DMSO
Baf. A1
Rel. p62 levels
tTA cont.
Thbs1 DTG
d
tTA cont.
Thbs1 DTG
WGA; LC3b
e
tTA cont.
Thbs1 DTG
1 µm
f
Adβgal
AdThbs1
MW (kDa)
Thbs1
Gapdh
-150
-37
g
Adβgal
AdThbs2
MW (kDa)
Thbs2
Gapdh
-150
-37
h
Adβgal
AdThbs3
MW (kDa)
Thbs3
Gapdh
-150
-37
i
tTA cont.
Thbs1 DTG
Thbs3 DTG
MW (kDa)
p-P70S6K
P70S6K
p-mTOR
mTOR
p-AKT
AKT
Gapdh
-75
-75
-250
-250
-50
-50
-37

Supplementary Figure 3. **Markers of autophagy and AKT/mTOR pathway in Thbs1 overexpressing hearts.**

a-b, Relative protein levels of LC3b-II and p62 determined by Western blot analysis from hearts of tTA cont., Thbs1 DTG and Thbs3 DTG mice and as shown in Fig. 5b at 4 weeks of age (n=3 biologically independent animals per group). *P =0.0002 versus tTA. cont. #P=0.0003 versus Thbs1 DTG for panel "a", and *P =0.0002 versus tTA. cont. #P=0.0002 versus Thbs1 DTG for panel "b". Statistical analysis was performed using one-way ANOVA and Tukey multiple comparisons test. Error bars are +/− standard error of the mean. **c**, Quantitative analysis of p62 protein levels relative to Gapdh from hearts of tTA cont., Thbs1 DTG treated with DMSO as vehicle or bafilomycin A1 (Baf. A1) treated to inhibit autolysosome degradation at 6 weeks of age (n=6 biologically independent animals per group). Data are represented as fold change compared to tTA cont. treated with DMSO; *P*-values are shown in the graph. Statistical analysis was performed using one-way ANOVA and Tukey multiple comparisons test. Error bars are +/− standard error of the mean. **d**, Higher magnification immunohistochemistry for LC3b protein (green) and WGA (purple) on heart sections of tTA cont. and Thbs1 DTG mice at 8 weeks of age, which shows LC3b in vesicles. Scale bars are 50 µm. **e**, Additional transmission electron microscopy (TEM) micrographs revealing autophagosomes (arrows) and autolysosomes (arrowheads) in heart sections of Thbs1 DTG hearts as compared to heart sections of tTA cont. mice at 6 weeks of age. Scale bar is 1 µm. **f-h**, Representative Western blot analysis confirming overexpression of Thbs1, Thbs2 and Thbs3 in neonatal rat ventricular cardiomyocytes infected for 48 hours with the indicated recombinant adenoviruses and compared to βgal control. Gapdh serves as loading control. Western blots are representative of Thbs1, 2 and 3 overexpression established in Fig. 5g-i. **i**, Representative Western blots for phospho-P70S6K (Thr 389), total-P70S6K, phospho-mTOR (Ser 2448), total-mTOR, phospho-AKT (Ser 473) and total-AKT on protein extracts from heart tissue of tTA cont., Thbs1 DTG and Thbs3 DTG mice at 6 weeks of age. Gapdh serves as loading control. Source data are provided as a Source Data File.

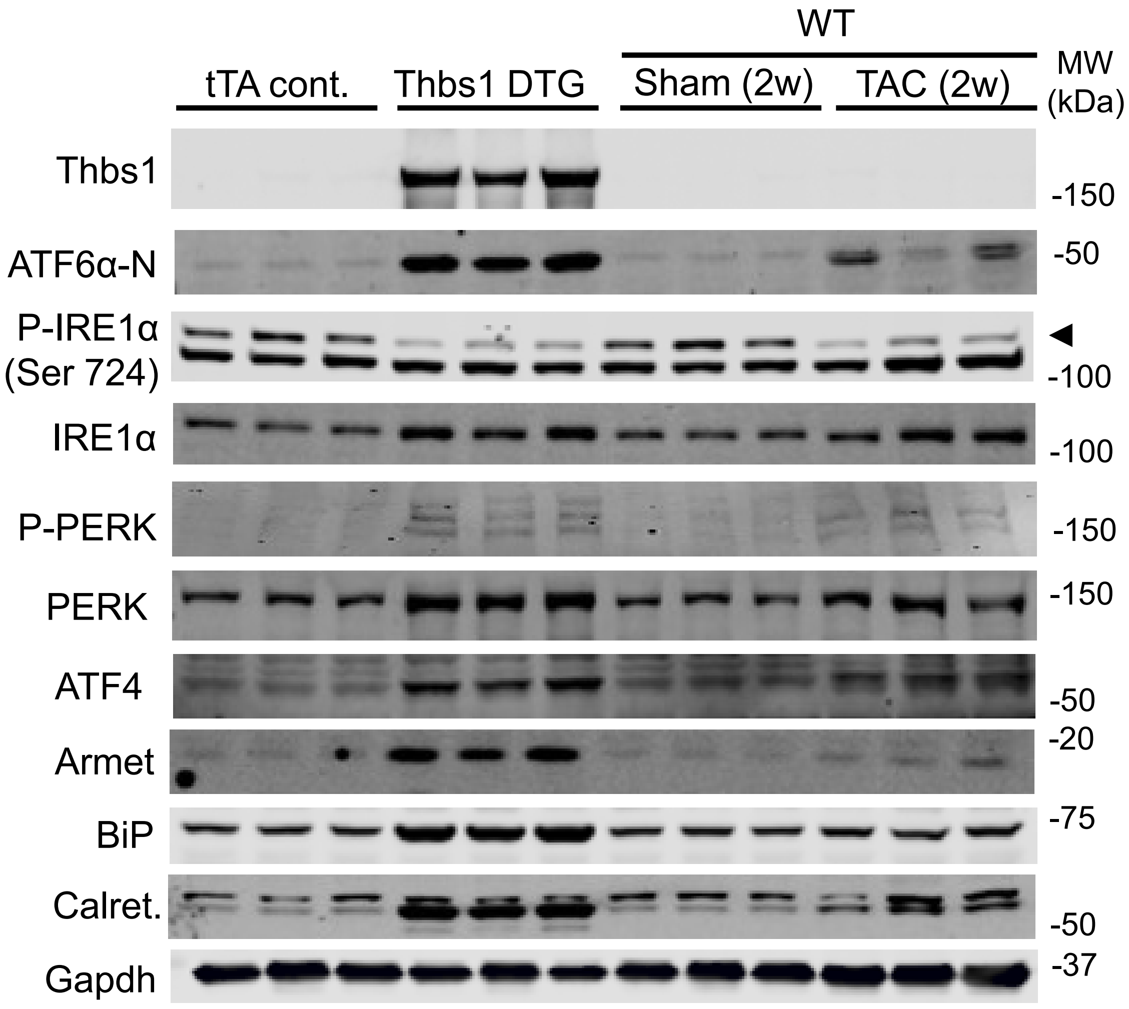

WT
tTA cont.
Thbs1 DTG
Sham (2w)
TAC (2w)
MW (kDa)
Thbs1
ATF6α-N
P-IRE1α (Ser 724)
IRE1α
P-PERK
PERK
ATF4
Armet
BiP
Calret.
Gapdh
-150
-50
-100
-100
-150
-150
-50
-20
-75
-50
-37

Supplementary Figure 4. **ER stress markers in Thbs1 transgenic and TAC-induced hypertrophic hearts**.

Representative Western blots for Thbs1, ATF6α-N (50 kDa, nuclear), phospho-IRE1α (Ser 724; arrowhead), total IRE1α, total PERK, ATF4, Armet, BiP, and calreticulin (Calret.). Phospho-PERK was determined with a Phos-tag gel. Blots were performed with protein extracts from heart tissue of tTA cont., Thbs1 DTG, sham-operated wildtype controls and mice that were subjected to 2 weeks of TAC at 8 weeks of age. Gapdh serves as loading control. Source data are provided as a Source Data File.

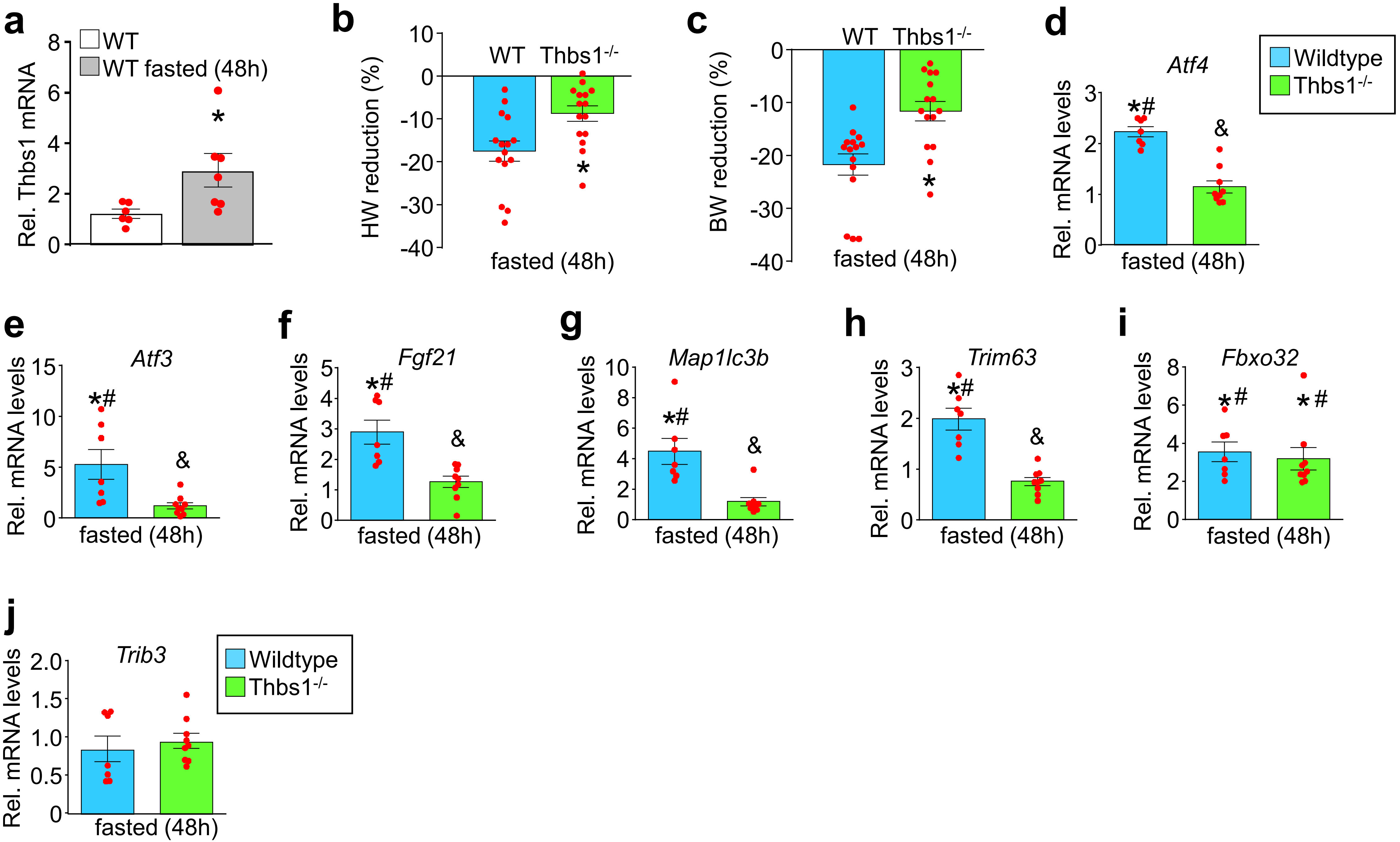
a
WT
WT fasted (48h)
Rel. Thbs1 mRNA
8
6
4
2
0
*
b
HW reduction (%)
10
0
-10
-20
-30
-40
WT
Thbs1-/-
*
fasted (48h)
c
BW reduction (%)
0
-10
-20
-30
-40
WT
Thbs1-/-
*
fasted (48h)
d
Atf4
Wildtype
Thbs1-/-
Rel. mRNA levels
3
2
1
0
*#
&
fasted (48h)
e
Atf3
Rel. mRNA levels
15
10
5
0
*#
&
fasted (48h)
f
Fgf21
Rel. mRNA levels
5
4
3
2
1
0
*#
&
fasted (48h)
g
Map1lc3b
Rel. mRNA levels
10
8
6
4
2
0
*#
&
fasted (48h)
h
Trim63
Rel. mRNA levels
3
2
1
0
*#
&
fasted (48h)
i
Fbxo32
Rel. mRNA levels
8
6
4
2
0
*#
*#
fasted (48h)
j
Trib3
Rel. mRNA levels
2.0
1.5
1.0
0.5
0
Wildtype
Thbs1-/-
fasted (48h)

Supplementary Figure 5. **Loss of *Thbs1* blunts fasting-induced cardiac atrophy.**

a, Quantitative RT-PCR results of Thbs1 mRNA from hearts of 8-week-old wildtype mice fed ad libitum or fasted for 48 hours (n=6 or 7 biologically independent animals, respectively). **P*=0.0432 versus wildtype. **b**, Percentage heart weight (HW) reduction after 48 hours fasting in wildtype and *Thbs1*$^{-/-}$ mice at 8 weeks of age. **P*=0.0064 versus wildtype. **c**, Percentage body weight (BW) reduction after 48 hours fasting in wildtype and *Thbs1*$^{-/-}$ mice. **P*=0.0010 versus wildtype. Statistical analysis was performed using two-tailed student's t-test. For "b and c", n=15 biologically independent animals per group. Error bars are +/− standard error of the mean. **d-j**, Quantitative RT-PCR results for *Atf4* (**P*<0.0001 vs fed wildtype, #*P*<0.0001 vs fed *Thbs1*$^{-/-}$,$^{\&}$*P*<0.0001 vs fasted wildtype), *Atf3* (**P*=0.0042 vs fed wildtype, #*P*=0.0041 vs fed *Thbs1*$^{-/-}$,$^{\&}$*P*=0.0045 vs fasted wildtype), *Fgf21* (**P*=0.0018 vs fed wildtype, #*P*=0.0018 vs fed *Thbs1*$^{-/-}$,$^{\&}$*P*=0.0050 vs fasted wildtype), *Map1lc3b* (LC3b; **P*=0.0020 vs fed wildtype, #*P*=0.0077 vs fed *Thbs1*$^{-/-}$,$^{\&}$*P*=0.0024 vs fasted wildtype), *Trim63* (MuRF1; **P*=0.0074 vs fed wildtype, #*P*=0.0072 vs fed *Thbs1*$^{-/-}$,$^{\&}$*P*=0.0004 vs fasted wildtype), *Fbxo32* (Atrogin-1; for fasted wildtype, **P*=0.0125 vs fed wildtype, #*P*=0.0196 vs fed *Thbs1*$^{-/-}$; for fasted *Thbs1*$^{-/-}$, **P*=0.0263 vs fed wildtype, #*P*=0.0417 vs fed *Thbs1*$^{-/-}$) and *Trib3* mRNA isolated from hearts of wildtype and *Thbs1*$^{-/-}$ mice after 48 hours fasting at 8 weeks of age. Data are represented as fold expression over fed wildtype; n=7 and 9 of biologically independent animals analyzed for wildtype and *Thbs1*$^{-/-}$ fasted, respectively. Statistical analysis was performed using a using two-tailed student's t-test for panels ”a-c”, and one-way ANOVA and Tukey multiple comparisons test for panels "d-j". All error bars are +/− standard error of the mean. Source data are provided as a Source Data File.

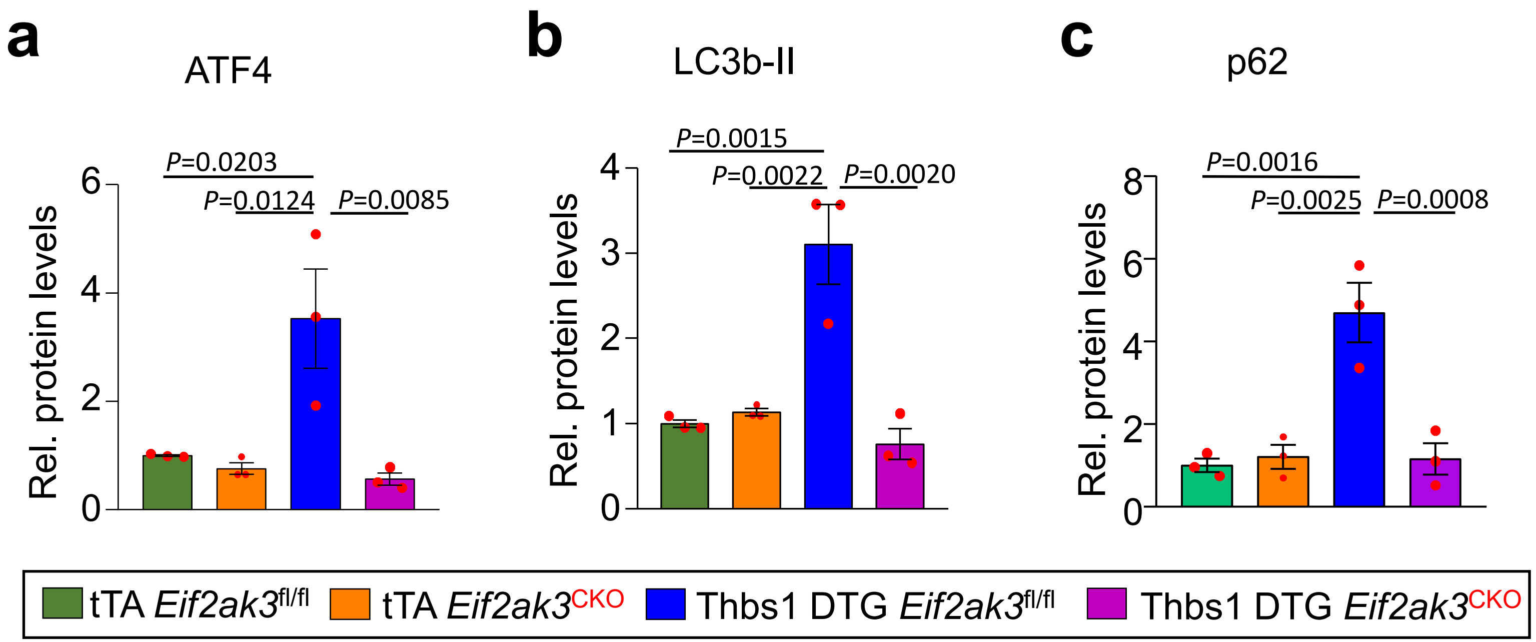

Supplementary Figure 6. **Deletion of *Eif2ak3* antagonizes Thbs1-mediated induction of ATF4, LC3b-II and p62.**

a-c, Quantitation of Western blots for ATF4, LC3b-II, and p62 shown in Fig. 7f (n=3 biologically independent animals per group). *P*-values are shown on the graphs. Statistical analysis was performed using one-way ANOVA and Tukey multiple comparisons test. Error bars are +/− standard error of the mean. Source data are provided as a Source Data File.

Supplementary Figure 7

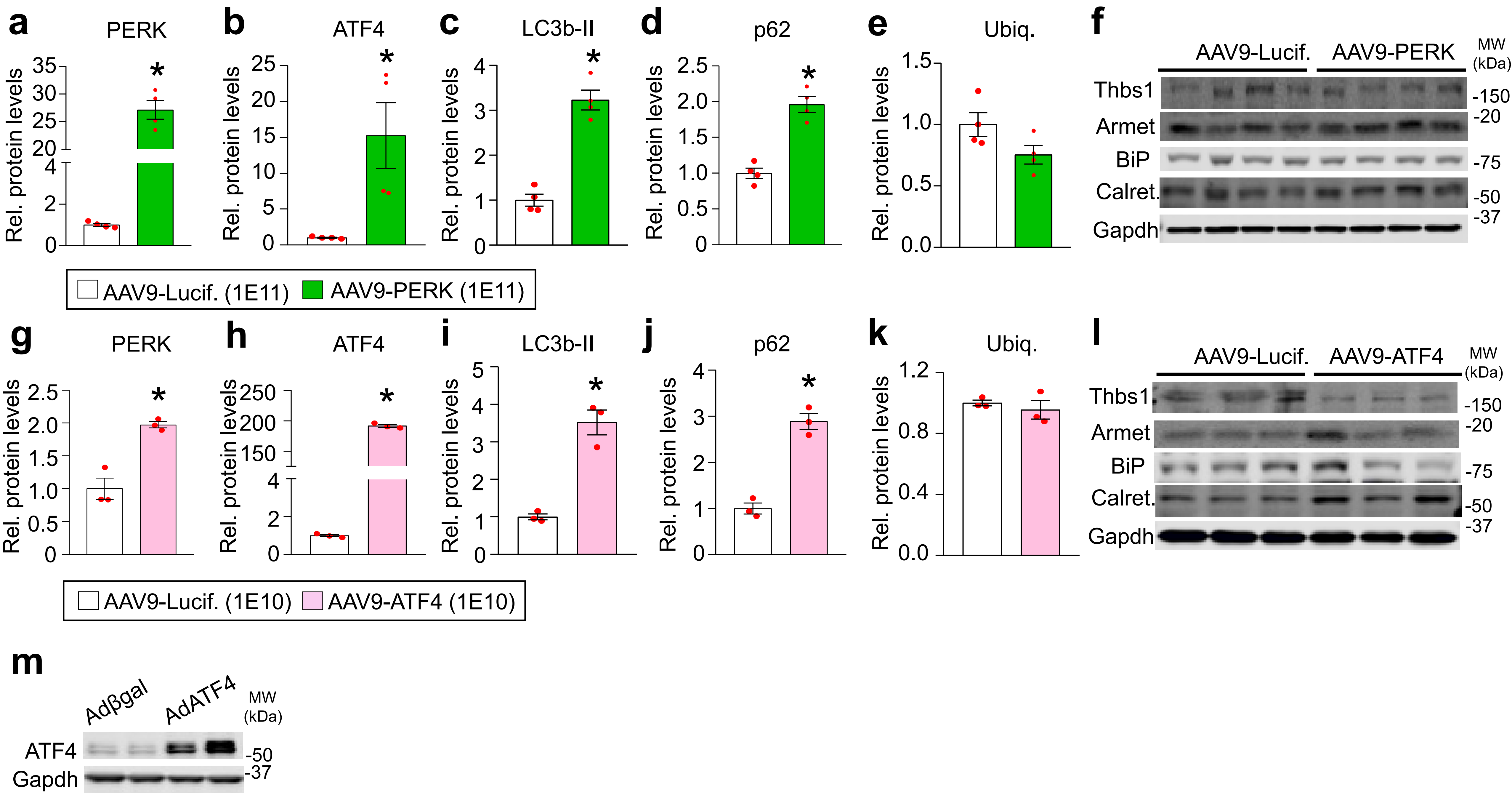

Supplementary Figure 7. **PERK and ATF4 are sufficient to induce cardiac atrophy.**

a-e, Relative protein levels from Western blots for PERK (**P*<0.0001), ATF4 (**P*=0.0205), LC3b-II (**P*=0.0001), p62 (**P*=0.0003) and ubiquitin-conjugated (Ubiq.; **P*=0.0919) proteins from cardiac protein extracts of 8-week-old mice injected with 1E11 genomic copies (gc) of AAV9-PERK or AAV9-Lucif. control and shown in Fig. 8c, d (n=4 biologically independent animals per group). Statistical analysis was performed using two-tailed student's t-test. Error bars are +/− standard error of the mean. **f**, Representative Western blots for Thbs1, Armet, BiP, and calreticulin (calret.) from cardiac protein extracts of 8-week-old mice injected with 1E11 gc of AAV9-PERK or AAV9-Lucif. control. Gapdh serves as a loading control. **g-k**, Relative protein levels of Western blots for PERK (**P*=0.0046), ATF4 (**P*<0.0001), LC3b-II (**P*=0.0018), p62 (**P*=0.0008) and ubiquitin-conjugated (Ubiq.) proteins from cardiac protein extracts of 4-week-old mice injected with 1E10 gc of AAV9-ATF4 or AAV9-Lucif. control and shown in Fig. 9b and 9j (1E10 gc, n=3 biologically independent animals per group). Statistical analysis was performed using two-tailed student's t-test. Error bars are +/− standard error of the mean. **l**, Representative Western blots for Thbs1, Armet, BiP, and calreticulin (calret.) from cardiac protein extracts of 4-week-old mice injected with 1E10 gc of AAV9-ATF4 or AAV9-Lucif. control. Gapdh serves as a loading control. **m**, Representative Western blot analysis confirming adenoviral-mediated overexpression of ATF4 in neonatal rat ventricular cardiomyocytes, 48 hours after infection with either AdATF4 or Adβgal control. Gapdh serves as loading control. Western blot is representative of ATF4 overexpression established in Fig. 9k,l. Source data are provided as a Source Data File.

Supplementary Table 1. **Cloning Primers**

**Target**	**Function**	**Sequence (5’-3’)**
α-MHC *Thbs1*	Forward	CATGTCGACATGGAGCTCCTGCGGGGACTAGGTGTC
	Reverse	GAGAAGCTTTAGGAATCTCGACACTCGTATTTCATGTC
α-MHC *Thbs1Δt1*	Forward	GGACAGGCATCCATCAATAGCAGAGTCGCTGGGCCAGCA
	Reverse	CCCAGCGACTCTGCTATTGATGGATGCCTGTCCAATC
α-MHC *Thbs2*	Forward	GTCGACATGCTCTGGGCACTGGCC
	Reverse	GTCGACCTAGGCATCTCTGCACTCATACTTG
pAAV-*Atf4*	Forward	AATTGGGATTCGAACATCGATATGACCGAGATGAGC
	Reverse	ACCCGTAGATCTCTCGAGTTACGGAACTCTCTT
pAAV-*Luciferase*	Forward	TGGGATTCGAACATCGATATGGAAGACGCCAAA
	Reverse	ACCCGTAGATCTCTCGAGTTACACGGCGATCTT

Supplementary Table 1. **qPCR Primers**

**Target**	**Forward Primer (5'-3')**	**Reverse Primer (5'-3')**
Manf	GACAGCCAGATCTGTGAACTAAAA	TTTCACCCGGAGCTTCTTC
Atf3	AAAAGGGGTGATGCAACG	TTAGCCGATTGGCTCCAC
Atf4	ATGATGGCTTGGCCAGTG	CCATTTTCTCCAACATCCAATC
Atf6	GGACGAGGTGGTGTCAGAG	GACAGCTCTTCGCTTTGGAC
Fbxo32	AGTGAGGACCGGCTACTGTG	GATCAAACGCTTGCGAATCT
Hspa5	CTGAGGCGTATTTGGGAAAG	TCATGACATTCAGTCCAGCAA
Calr	TGAAGCTGTTTCCGAGTGGT	GATGACATGAACCTTCTTGGTG
Ddit3	CCTAGCTTGGCTGACAGAGG	CTGCTCCTTCTCCTTCATGC
Fgf21	AGATGGAGCTCTCTATGGATCG	GGGCTTCAGACTGGTACACAT
Ern1	ACACCGACCACCGTATCTCA	CTCAGGATAATGGTAGCCATGTC
Map1lc3b	CAGTGTCAGGGGCAGTCTC	TGAGTGGGAGCCCTTTTAGA
Trim63	AGAGTGAGCTGAGCGATGG	GTCTGCGGCTGTTGTCCT
Eif2ak3	CGAGGGACACTCCTTTGAAC	AGGAGGACGTTCCTTCCCTA
Thbs1	GGGGAGATAACGGTGTGTTTG	CGGGGATCAGGTTGGCATT
Trib3	CGCTTTGTCTTCAGCAACTGT	TCATCTGATCCAGTCATCACG
Rpl13	GCCGGACTCCCTACAAGC	GCTTCAGTATCATGCCATTCC
