# Supplementary material for: Thbs1 induces lethal cardiac atrophy through PERK-ATF4 regulated autophagy
Source: Nat Commun. 2021 Jun 24;12:3928. doi: 10.1038/s41467-021-24215-4 (PMC8225674; doi:10.1038/s41467-021-24215-4)
Supplement: Supplementary file 2 — Reporting Summary [file 41467_2021_24215_MOESM2_ESM.pdf]

nature research

Corresponding author(s): Jeffery D. Molkentin

Last updated by author(s): May 18, 2021

Reporting Summary

Nature Research wishes to improve the reproducibility of the work that we publish. This form provides structure for consistency and transparency in reporting. For further information on Nature Research policies, see our Editorial Policies and the Editorial Policy Checklist.

Statistics

For all statistical analyses, confirm that the following items are present in the figure legend, table legend, main text, or Methods section.

n/a	Confirmed	
☐	☒	The exact sample size (n) for each experimental group/condition, given as a discrete number and unit of measurement
☐	☒	A statement on whether measurements were taken from distinct samples or whether the same sample was measured repeatedly
☐	☒	The statistical test(s) used AND whether they are one- or two-sided<br>*Only common tests should be described solely by name; describe more complex techniques in the Methods section.*
☐	☒	A description of all covariates tested
☐	☒	A description of any assumptions or corrections, such as tests of normality and adjustment for multiple comparisons
☐	☒	A full description of the statistical parameters including central tendency (e.g. means) or other basic estimates (e.g. regression coefficient) AND variation (e.g. standard deviation) or associated estimates of uncertainty (e.g. confidence intervals)
☐	☒	For null hypothesis testing, the test statistic (e.g. F, t, r) with confidence intervals, effect sizes, degrees of freedom and P value noted<br>*Give P values as exact values whenever suitable.*
☒	☐	For Bayesian analysis, information on the choice of priors and Markov chain Monte Carlo settings
☒	☐	For hierarchical and complex designs, identification of the appropriate level for tests and full reporting of outcomes
☒	☐	Estimates of effect sizes (e.g. Cohen's d, Pearson's r), indicating how they were calculated

Our web collection on statistics for biologists contains articles on many of the points above.

Software and code

Policy information about availability of computer code

Data collection

Data collection procedures are described in the Methods section.
Software used:
GraphPad Prism: GraphPad Prism 9.0.0 Software (www.graphpad.com)
NIS-Elements Advanced Research (AR) microscope imaging software: NIS Elements AR 5.02.01 64 bit software (www.nikoninstruments.com)
Leica Application Suite Leica Microsystems: LAS X software (www.leica-microsystems.com)
Odyssey CLx imaging system with Li-Cor Image Studio version 5.2 software (www.licor.com)
HP Sonos 5500 Ultrasound System (Ref M2424A) with 2004 version D.2. software (www.somatechnology.com)
Bio-Rad CFX96 C1000 Touch Real-Time PCR System with BIORAD CFX Maestro 1.1 (4.1.233.1219) software (www.bio-rad.com)

Data analysis

Data collection procedures are described in the Methods section.
Software used:
GraphPad Prism: GraphPad Prism 9.0.0 Software www.graphpad.com
NIS-Elements Advanced Research (AR) microscope imaging software: NIS Elements AR 5.02.01 64 bit software www.nikoninstruments.com
Leica Application Suite Leica Microsystems : LAS X software www.leica-microsystems.com
Odyssey CLx imaging system with Li-Cor Image Studio version 5.2 software (www.licor.com)
HP Sonos 5500 Ultrasound System (Ref M2424A) with 2004 version D.2. software (www.somatechnology.com)
Bio-Rad CFX96 C1000Touch Real-Time PCR System with BIORAD CFX Maestro 1.1 (4.1.233.1219) software (www.bio-rad.com)

For manuscripts utilizing custom algorithms or software that are central to the research but not yet described in published literature, software must be made available to editors and reviewers. We strongly encourage code deposition in a community repository (e.g. GitHub). See the Nature Research guidelines for submitting code & software for further information.

Data

Policy information about availability of data

All manuscripts must include a data availability statement. This statement should provide the following information, where applicable:

- Accession codes, unique identifiers, or web links for publicly available datasets
- A list of figures that have associated raw data
- A description of any restrictions on data availability

All original data underlying selected data shown in the figures and supplemental figures are available from the corresponding author upon reasonable request.

Field-specific reporting

Please select the one below that is the best fit for your research. If you are not sure, read the appropriate sections before making your selection.

☒ Life sciences ☐ Behavioural & social sciences ☐ Ecological, evolutionary & environmental sciences

For a reference copy of the document with all sections, see nature.com/documents/nr-reporting-summary-flat.pdf

Life sciences study design

All studies must disclose on these points even when the disclosure is negative.

Sample size	The n numbers used in this study reflects the minimum number needed to achieve statistical significance based on experience and previous power analysis. Sample sizes were chosen based on extensive prior experience of the lab in characterizing cardiac injury models in the mouse (including observed post-operative surgical mortality rates for TAC surgery). These studies include -but are not limited too- reference 9 (Schips, T.G. et al. nat Commun 2019), reference 13 (Lynch, J.M. et al. Cell 2012), reference 20 (Correll, R.N. et al. Sci Rep 2019) and reference 50 (Accornero, F. et al. Mol Cell Biol 2015) of this study.
Data exclusions	Mice with pressure gradients of less than 45 mmHg after TAC were excluded from the results, as this indicated an unsuccessful surgery. Animals that did not survive the day of surgery were excluded. These exclusions apply to the TAC-associated data presented in figure 1c, figure 6a-p, and supplementary figure 4 of this study.
Replication	The results of all in vivo experiments were reproducible as shown across multiple animals (exact n values indicated in the text and figures) over multiple surgical cohorts. In vitro experimental findings were independently reproduced at least 3 times (exact n values indicated in the text and figures) and replication was successful.
Randomization	The animals were not randomized because they were genetically identical within groups. Both sexes of mice were used.
Blinding	Blinding was performed for most experimental procedures, although blinding was not possible in every instance. Surgeries and cardiac injections, as well as analysis of echocardiographic data, all qPCR and histological analyses were performed by investigators blinded to genetic background of the mice, experimental treatment or procedure. However, in order to perform Western blot analysis the investigator needed to know in which order the samples needed to be loaded on the acylamide gels and then blinding was not possible.

Reporting for specific materials, systems and methods

We require information from authors about some types of materials, experimental systems and methods used in many studies. Here, indicate whether each material, system or method listed is relevant to your study. If you are not sure if a list item applies to your research, read the appropriate section before selecting a response.

Materials & experimental systems

n/a	Involved in the study	
☐	☒	Antibodies
☒	☐	Eukaryotic cell lines
☒	☐	Palaeontology and archaeology
☐	☒	Animals and other organisms
☒	☐	Human research participants
☒	☐	Clinical data
☒	☐	Dual use research of concern

Methods

n/a	Involved in the study	
☒	☐	ChIP-seq
☒	☐	Flow cytometry
☒	☐	MRI-based neuroimaging

Antibodies

Antibodies used	Rabbit anti-Armet Abcam, Cat #ab67271;<br>Rabbit anti-p-AKT ser473 Cell Signaling Technology, Cat #9271;<br>Rabbit anti-AKT ser473 Cell Signaling Technology, Cat #9272;

Rabbit anti-ATF4 Cell Signaling Technology, Cat #11815;
Rabbit anti-ATF6α SAB SignalWayAntibody LLC, #SAB24383;
Rabbit anti-calreticulin (CRT) Cell Signaling Technology, Cat #2891;
Rat anti-CD31 BD Biosciences, Cat #553370;
Rabbit anti-CD36 Abacm, Cat #ab133625;
Rabbit anti-CD47 Abacm, Cat #ab108415;
Rabbit anti-Derlin-1 Abcam, #ab176732;
Rabbit anti-Derlin-3 Sigma, #D2194;
Rabbit anti-DNAJ3 Abcam, Cat #ab227140;
Rabbit anti-Edem1 LifeSpan Biosciences, #LS-C80983;
Rabbit anti-p-eIF2a Ser51 Cell Signaling Technology, Cat #9721;
Rabbit anti-eIF2a Cell Signaling Technology, Cat #9722;
Rabbit anti-GCN2 Abcam, Cat #ab134053;
Rabbit anti-p-GCN2 thr667 Abcam, #ab68427;
Rabbit anti-Herpud1 Cell Signaling Technology, #26730;
Rabbit anti-Ire1α Cell Signaling Technology, #3294;
Rabbit anti-p-Ire1α ser724 Abcam, #ab48187;
Rabbit anti-GRP78/BiP Sigma-Aldrich Cat #G8918;
Rabbit anti-Lc3b Cell Signaling Technology, Cat #3868;
Rabbit anti-mTOR Cell Signaling Technolgy, Cat #2972;
Rabbit anti-p62 Sigma, Cat #p0067;
Rabbit anti-p-mTOR ser2448 Cell Signaling Technolgy, Cat #2971;
Rabbit anti-p-p70S6K Thr389 Cell Signaling Technology, Cat #9205;
Rabbit anti-p70S6K Cell Signaling Technology, Cat #9202;
Rabbit anti-Perk antibody Proteintech, Cat #20582-1-AP;
Rabbit anti-PERK Cell Signaling Technology, Cat #3192;
Rabbit anti-PKR Abcam, Cat #ab184257;
Rabbit anti-SEL1L Sigma, Cat #S3699;
Goat anti-Thbs1 R&D Systems, Cat #AF3074;
Mouse anti-Thbs1 Invitrogen, Cat #MA5-13395;
Mouse anti-Thbs2 BD Bioscience Cat #611150;
Mouse anti-Thbs2 R&D Systems, #MAB1635;
Rabbit anti-Thbs3 Proteintech Cat #19727-1-AP;
Mouse anti-alpha tubulin Sigma, Cat #T5168;
Mouse anti-Ubiquitin Santa Cruz Biotechnology, Cat # sc-8017;
Rabbit anti-VCP/P97 Abcam, Cat #ab36047;
Mouse anti-Vinculin Sigma, Cat #V9131;
Rabbit anti Vimentin Abcam, Cat#ab45939;
Biotinylated GSL I Isolectin B4 Vector Laboratories, Cat #B-1205;
Mouse anti Gapdh Fitzgerald, Cat # 10R-G109A;

Goat anti-Mouse IRdye 800CW LI-COR, Cat #926-32350
Goat anti-Rat IRdye 800CW LI-COR, Cat #926-32219
Goat anti-Rabbit IRdye 800CW LI-COR, Cat #926-32211
Goat anti-Mouse IRdye 680RD LI-COR, Cat #926-68072
Goat anti-Rabbit IRdye 680RD LI-COR, Cat #925-68073
Donkey anti-Goat IRdye 800CW LI-COR, Cat #926-32214
Donkey anti-Goat IRdye 680RD LI-COR, Cat #926-68074
Donkey anti-Mouse IRdye 680RD LI-COR, Cat #926-68072
Goat anti-Mouse Alexa Fluor-488 ThermoFisher Scientific, Cat #A11029
Goat anti-Rabbit Alexa Fluor-488 ThermoFisher Scientific, Cat #A11008
Donkey anti-Goat Alexa Fluor--488 ThermoFisher Scientific, Cat #A11055
Goat anti-Mouse Alexa Fluor-568 ThermoFisher Scientific, Cat #A11031
Goat anti-Rabbit Alexa Fluor-568 ThermoFisher Scientific, Cat #A11036
Goat anti-Rat Alexa Fluor-568 ThermoFisher Scientific, Cat #A11077
Donkey anti-Rabbit Alexa Fluor-568 ThermoFisher Scientific, Cat #A10042
Donkey anti-Goat Alexa Fluor--568 ThermoFisher Scientific, Cat #A11057
Streptavidin Alexa-594 conjugate ThermoFisher Scientific, Cat #S11227

Validation

All antibodies used are commercially available and have been characterized by the manufacturers (and most of them in several publications) for their reactivity in the appropriate species and for their compatibility to be used with the respective application. In addition, for Western blot we used molecular weight markers to identify the band(s) that migrated at the expected size of each respective protein analyzed, and used overexpression or knockout samples whenever possible. Staining of samples in this study was also compared to unstained or secondary antibody-only controls processed in the same way. As indicated below validation details are also available on the manufacturers' websites.

1. General validation statements:

- Abcam: https://www.abcam.com/primary-antibodies/how-we-validate-our-antibodies

- Cell Signaling Technology: https://www.cellsignal.com/about-us/cst-antibody-performance-guarantee.
- Sigma: https://www.sigmaaldrich.com/life-science/cell-biology/antibodies/antibody-validation.html
- LSBio: https://www.lsbio.com/resources/lsbio-advantage#lsbio-guarantee
- R&D Systems: https://www.rndsystems.com/quality/antibodies-built-for-reproducibility
- Vector labs: https://vectorlabs.com/browse/antibodies

2. Antibodies used in this study:

- Rabbit anti-Armet Abcam, Cat #ab67271:
This antibody was knockout validated by the manufacturer and cited in 19 papers on the manufacturer's website.
Manufacturer's validation information can be found on: https://www.abcam.com/armetarp-antibody-ab67271.html

- Rabbit anti-p-AKT ser473 Cell Signaling Technology, Cat #9271:
This antibody is cited in >4550 papers on the manufacturer's website.
Manufacturer's validation information can be found on: https://www.cellsignal.com//products/primary-antibodies/phospho-akt-ser473-antibody/9271

- Rabbit anti-AKT ser473 Cell Signaling Technology, Cat #9272:
This antibody is cited in >5800 papers on the manufacturer's website.
Manufacturer's validation information can be found on: https://www.cellsignal.com//products/primary-antibodies/akt-antibody/9272

- Rabbit anti-ATF4 Cell Signaling Technology, Cat #11815:
This antibody is cited in >300 papers on the manufacturer's website.
Manufacturer's validation information can be found on: https://www.cellsignal.com//products/primary-antibodies/atf-4-d4b8-rabbit-mab/11815
In addition, this antibody was validated for Western blot analysis by adenoviral mediated overexpression of ATF4 in neonatal rat cardiomyocytes (see Supplementary Figure 7m).

- Rabbit anti-ATF6α SAB SignalWayAntibody LLC, #SAB24383:
This antibody was knockout validated in our lab (Correll, R.N. et al. Scientific Reports 2019)
Manufacturer's information can be found on: https://www.sabbiotech.com/g-2410-ATF6-Antibody-24383.html

- Rabbit anti-calreticulin (CRT) Cell Signaling Technology, Cat #2891:
This antibody is cited in 50 papers on the manufacturer's website.
Manufacturer's validation information can be found on: https://www.cellsignal.com//products/primary-antibodies/calreticulin-antibody/2891

- Rat anti-CD31 BD Biosciences, Cat #553370:
Manufacturer's validation information: http://www.bdbiosciences.com/ds/pm/tds/553370.pdf

- Rabbit anti-CD36 Abcam, Cat #ab133625:
This antibody is cited in 68 papers on the manufacturer's website. In addition, our study validated this antibody by Western blot analysis using protein homogenates of CD36-null hearts (Supplementary Figure 1d)
Manufacturer's validation information can be found on: https://www.abcam.com/cd36-antibody-epr6573-ab133625.html

- Rabbit anti-CD47 Abcam, Cat #ab108415:
Current study validated this antibody by Western blot analysis using protein homogenates of CD47-null hearts (Supplementary Figure 1a)
Manufacturer's validation information can be found on: https://www.abcam.com/cd47-antibody-epr41502-ab108415.html

- Rabbit anti-Derlin-1 Abcam, #ab176732:
This antibody is cited in 1 paper on the manufacturer's website.
Manufacturer's validation information can be found on: https://www.abcam.com/derl1derlin-1-antibody-ab176732.html

- Rabbit anti-Derlin-3 Sigma, #D2194:
This antibody is cited in 4 papers on the manufacturer's website.
Manufacturer's validation information can be found on: https://www.sigmaaldrich.com/catalog/product/sigma/d2194?lang=en&region=US

- Rabbit anti-DNAJ3 Abcam, Cat #ab227140:
Manufacturer's validation information can be found on: https://www.abcam.com/dnajc3-antibody-ab227140.html

- Rabbit anti-Edem1 LifeSpan Biosciences, #LS-C80983:
Manufacturer's validation information can be found on: https://www.lsbio.com/antibodies/edem1-antibody-edem-antibody-aa146-195-wb-western-ls-c80983/81612#specifications-section

- Rabbit anti-p-eIF2a Ser51 Cell Signaling Technology, Cat #9721:
This antibody is cited in >550 papers on the manufacturer's website.
Manufacturer's validation information can be found on: https://www.cellsignal.com//products/primary-antibodies/phospho-eif2a-ser51-antibody/9721

- Rabbit anti-eIF2a Cell Signaling Technology, Cat #9722:
This antibody is cited in >490 papers on the manufacturer's website.
Manufacturer's validation information can be found on: https://www.cellsignal.com//products/primary-antibodies/eif2a-antibody/9722

- Rabbit anti-GCN2 Abcam, Cat #ab134053:
This antibody was knockout validated by the manufacturer and is cited in 5 papers on the manufacturer's website. Manufacturer's validation information can be found on:
https://www.abcam.com/gcn2-antibody-epr59702-ab134053.html

- Rabbit anti-p-GCN2 thr667 Abcam, #ab68427:
Manufacturer's validation information can be found on: https://www.abcam.com/gcn2-phospho-t667-antibody-epr2319y-ab68427.html

- Rabbit anti-Herpud1 Cell Signaling Technology, #26730:
This antibody is cited in 1 paper on the manufacturer's website.
Manufacturer's validation information can be found on: https://www.cellsignal.com//products/primary-antibodies/herpud1-antibody/26730

- Rabbit anti-Ire1α Cell Signaling Technology, #3294:
This antibody is cited in >400 papers on the manufacturer's website.
Manufacturer's validation information can be found on: https://www.cellsignal.com//products/primary-antibodies/ire1a-14c10-rabbit-mab/3294

- Rabbit anti-p-Ire1α ser724 Abcam, #ab48187:
This antibody is cited in >170 papers on the manufacturer's website.
Manufacturer's validation information can be found on: https://www.abcam.com/ire1-phospho-s724-antibody-ab48187.html

- Rabbit anti-GRP78/BiP Sigma-Aldrich Cat #G8918:
This antibody is cited in >890 papers on the manufacturer's website and has been routinely used in our lab.
Manufacturer's validation information can be found on: https://www.sigmaaldrich.com/catalog/product/sigma/g8918?lang=en&region=US

- Rabbit anti-Lc3b Cell Signaling Technology, Cat #3868:
This antibody is cited in >890 papers on the manufacturer's website.
Manufacturer's validation information can be found on: https://www.cellsignal.com//products/primary-antibodies/lc3b-d11-xp-rabbit-mab/3868

- Rabbit anti-mTOR Cell Signaling Technolgy, Cat #2972:
This antibody is cited in >1000 papers on the manufacturer's website.
Manufacturer's validation information can be found on: https://www.cellsignal.com//products/primary-antibodies/mtor-antibody/2972

- Rabbit anti-p62 Sigma, Cat #p0067:
This antibody is cited in >420 papers on the manufacturer's website.
Manufacturer's validation information can be found on: https://www.sigmaaldrich.com/catalog/product/sigma/p0067?lang=en&region=US

- Rabbit anti-p-mTOR ser2448 Cell Signaling Technolgy, Cat #2971;
This antibody is cited in >1200 papers on the manufacturer's website.
Manufacturer's validation information can be found on: https://www.cellsignal.com//products/primary-antibodies/phospho-mtor-ser2448-antibody/2971

- Rabbit anti-p-p70S6K Thr389 Cell Signaling Technology, Cat #9205;
This antibody is cited in >1100 papers on the manufacturer's website.
Manufacturer's validation information can be found on: https://www.cellsignal.com//products/primary-antibodies/phospho-p70-s6-kinase-thr389-antibody/9205

- Rabbit anti-p70S6K Cell Signaling Technology, Cat #9202;
This antibody is cited in >1100 papers on the manufacturer's website.
Manufacturer's validation information can be found on: https://www.cellsignal.com//products/primary-antibodies/p70-s6-kinase-antibody/9202

- Rabbit anti-Perk antibody Proteintech, Cat #20582-1-AP:
This antibody was knockout validated by the manufacturer and is cited in 35 papers on the manufacturer's website.

Manufacturer's validation information can be found on:
https://www.ptglab.com/products/PERK-Antibody-20582-1-AP.htm

- Rabbit anti-PERK Cell Signaling Technology, Cat #3192:
This antibody is cited in >300 papers on the manufacturer's website. Furthermore, our study provides additional knockout validation for this antibody for Western blotting in Figure 7f.
Manufacturer's validation information can be found on: https://www.cellsignal.com//products/primary-antibodies/perk-c33e10-rabbit-mab/3192

- Rabbit anti-PKR Abcam, Cat #ab184257:
This antibody was knockout validated by the manufacturer and is cited in 2 papers on the manufacturer's website.
Manufacturer's validation information can be found on: https://www.abcam.com/pkr-antibody-epr19374-ab184257.html

- Rabbit anti-SEL1L Sigma, Cat #S3699:
This antibody was knockout validated by the manufacturer and is cited in 15 papers on the manufacturer's website.
Manufacturer's validation information can be found on: https://www.sigmaaldrich.com/catalog/product/sigma/s3699?lang=en&region=US

- Goat anti-Thbs1 R&D Systems, Cat #AF3074:
This antibody was knockout validated in our laboratory using protein extracts obtained from Thbs1-null heart and skeletal muscle as compared to wildtype control. In addition, this antibody is cited in 4 papers on the manufacturer's website.
Manufacturer's validation information can be found on: https://www.rndsystems.com/products/human-thrombospondin-1-antibody_af3074

- Mouse anti-Thbs1 Invitrogen, Cat #MA5-13395:
This antibody was knockout validated in our laboratory using protein extracts obtained from Thbs1-null heart and skeletal muscle as compared to wildtype control. In addition, this antibody is cited in 94 papers on the manufacturer's website.
Manufacturer's validation information can be found on: https://www.thermofisher.com/antibody/product/Thrombospondin-1-Antibody-clone-A6-1-Monoclonal/MA5-13395

Mouse anti-Thbs2 BD Bioscience Cat #611150:
This antibody was validated in our laboratory for western blot analysis in cardiac-specific Thbs2 overexpressing hearts (Figure 2k).
Manufacturer's validation information can be found on: https://www.bdbiosciences.com/us/reagents/research/antibodies-buffers/cell-biology-reagents/cell-biology-antibodies/purified-mouse-anti-mouse-thrombospondin-2-4thrombospondin-2/p/611150

- Mouse anti-Thbs2 R&D Systems, #MAB1635:
This antibody was validated in our laboratory by adenoviral mediated overexpression of Thbs2 in neonatal rat cardiomyocytes (SupFig. 3g).
Manufacturer's validation information can be found on: https://www.rndsystems.com/products/human-thrombospondin-2-antibody-230934_mab1635

- Rabbit anti-Thbs3 Proteintech Cat #19727-1-AP:
This antibody was previously knockout validated in our lab and published by Schips TG et al. Nature Communications 2019.
Manufacturer's validation information can be found on: https://www.ptglab.com/products/THBS3-Specific-Antibody-19727-1-AP.htm

- Mouse anti-alpha tubulin Sigma, Cat #T5168:
This antibody is cited in >2600 papers on the manufacturer's website.
Manufacturer's validation information can be found on: https://www.sigmaaldrich.com/catalog/product/sigma/t5168?lang=en&region=US

- Mouse anti-Ubiquitin Santa Cruz Biotechnology, Cat # sc-8017:
This antibody is cited in >60 papers on the manufacturer's website.
Manufacturer's validation information can be found on: https://www.scbt.com/p/ubiquitin-antibody-a-5?gclid=Cj0KCQjwytOEBhD5ARIsANnRjVi1h-hHJ_mPM9jKUvzYbMwbwRtyx6fsGlI39EJ5c8IxBsNmmG1Pv34aAuqCEALw_wcB

- Rabbit anti-VCP/P97 Abcam, Cat #ab36047:
This antibody is cited in 3 papers on the manufacturer's website.
Manufacturer's validation information can be found on: https://www.abcam.com/vcp-antibody-ab36047.html

- Mouse anti-Vinculin Sigma, Cat #V9131:
This antibody is cited in >1100 papers on the manufacturer's website.
Manufacturer's validation information can be found on:
https://www.sigmaaldrich.com/catalog/product/sigma/v9131?lang=en&region=US

- Rabbit anti Vimentin Abcam, Cat#ab45939:
This antibody was knockout validated and is cited in >1100 papers on the manufacturer's website. Manufacturer's validation information can be found on: https://www.abcam.com/vimentin-antibody-cytoskeleton-marker-ab45939.html

- Biotinylated GSL I Isolectin B4 Vector Laboratories, Cat #B-1205:
This antibody is cited in >320 papers on the manufacturer's website.

Manufacturer’s validation information can be found on:
https://vectorlabs.com/biotinylated-gsl-i-isolectin-b4.html

- Mouse anti Gapdh Fitzgerald, Cat # 10R-G109A:
This antibody is routinely used in our laboratory. Citations include -but are not limited too- reference 9 (Schips, T.G. et al. nat Commun 2019), reference 10 (Vanhoutte, D. et al. eLife 2016), reference 13 (Lynch, J.M. et al. Cell 2012), reference 20 (Correll, R.N. et al. Sci Rep 2019) and reference 50 (Accornero, F. et al. Mol Cell Biol 2015) of this study.
Manufacturer’s validation information can be found on: https://www.fitzgerald-fii.com/gapdh-antibody-10r-g109a.html

Animals and other organisms

Policy information about studies involving animals; ARRIVE guidelines recommended for reporting animal research

Laboratory animals	All mice included in this study were on a C57Bl/6J genetic background. Mouse strains used in this study include cardiomyocyte-specific Thbs1, 2, and 3 transgenic mice; mice deficient for Thbs1, Cd36, Cd47, and Eif2ak3-loxP targeted (B6.129S2-Thbs1tm1Hyn/J, B6.129S1-Cd36tm1Mfe/J, B6.129S7-Cd47tm1Fpl/J, and Eif2ak3tm1.2Drc/J); mice deficient for ATF6α; βMHC-Cre mice, cardiomyocyte-specific activated calcineurin overexpressing mice (delta-CnA TG) and mice deficient for Csrp3. All mice included in this study ranged from 4 to 32 weeks of age, pending the experiment. Both sexes of mice were used in equal ratios.
Wild animals	No wild animals were used in this study.
Field-collected samples	This study did not involve field-collected samples.
Ethics oversight	All experimental procedures with mice were approved by the Institutional Animal Care and Use Committee of Cincinnati Children’s Medical Center, protocols IACUC 2018-0047 and 2016-0069. We have complied with the relevant ethical considerations for animal usage overseen by this committee.

Note that full information on the approval of the study protocol must also be provided in the manuscript.
